# Immune activation of characteristic gut mycobiota *Kazachstania pintolopesii* on IL-23/IL-17R signaling in ankylosing spondylitis

**DOI:** 10.3389/fcimb.2022.1035366

**Published:** 2022-12-20

**Authors:** Haiting Zhang, Yu Wei, Huanhuan Jia, Diling Chen, Xiaocui Tang, Jian Wang, Meili Chen, Yinrui Guo

**Affiliations:** ^1^ Guangdong Second Provincial General Hospital, Guangzhou, Guangdong, China; ^2^ Guangzhou University of Chinese Medicine, Guangzhou, Guangdong, China; ^3^ Guangdong Key Laboratory of Laboratory Animals, Guangdong Laboratory Animals Monitoring Institute, Guangzhou, Guangdong, China; ^4^ Guangzhou Laboratory, Guangzhou, Guangdong, China; ^5^ Zhuhai Hospital of Integrated Traditional Chinese and Western Medicine, Zhuhai, Guangdong, China

**Keywords:** fungal and bacterial dysbiosis, communication/interaction network, autoimmune response, ankylosing spondylitis, *Kazachstania pintolopesii*

## Abstract

It is very important to understand the communication and interaction mechanisms between the host and its resident microorganisms on host physiology and for precise diagnosis and treatment. Although intestinal fungi and bacteria dysbiosis is increasingly linked to ankylosing spondylitis (AS), their mechanisms of action have been rarely illustrated. In this paper, fecal samples from 10 AS monkeys and 10 healthy controls were collected to systematically characterize the gut mycobiota and microbiota in AS monkeys by 16S rRNA and ITS2 DNA sequencing. Our results showed the gut fungi of *Kazachstania pintolopesii*, Saccharomycetaceae, Kazachstania, and Saccharomyceteles. Saccharomycetes were specially enriched in AS, and the microbiota of AS monkeys was characterized by an increased abundance of Clostridia, Clostridiales, Ruminococcaceae, and *Prevotella 2*, using Line Discriminant Analysis Effect Size. Compared to healthy controls, decreased ITS2/16S biodiversity ratios and altered bacterial–fungal interkingdom networks were observed in AS monkeys. Oral administration of *K. pintolopesii* activates IL-17RA pathway and induce inflammatory reaction in the colonic tissue of C57BL/6 mice, as well as multiple AS phenotypes, including fungal and bacterial dysbiosis, immune responses of NK cells, platelets, T cells, leukocytes, B-cell activation, rheumatoid arthritis, and inflammatory bowel disease. We also found the secreted products of *K. pintolopesii* could activate the IL-17RA pathway, which induces PANoptosis in macrophage RAW264.7 cells. Much worse, the PANoptosis products could promote the proliferation and morphological changes of *K. pintolopesii*, which resulted in much more *K. pintolopesii* and a severe inflammatory reaction. Interestingly, the inflammatory factor TNF-α can promote the morphological transformation of *Candida albicans* and *K. pintolopesii*, which is worthy of further study. The characteristic fungi in all these findings implied that fungal and bacterial dysbiosis have a close link to AS and that their communication and interaction indeed play an important role in autoimmune responses, and *K. pintolopesii* could be a potential marker microorganism in AS, although its specific mechanism is not fully elucidated.

## 1 Introduction

The importance of the intestinal microbiome for host homeostasis as well as the pathogenesis and treatment of a wide range of inflammatory, immune, chronic, and malignant diseases has become increasingly evident (Honda and Littman, 2016; Rooks and Garrett, 2016). So far, however, most studies have focused on the role of bacteria; another class of commensals, namely the mycobiota, might also contribute substantially, especially conditionally pathogenic fungi such as *Candida albicans.* A previous study revealed that the human anti-fungal Th17 immunity and pathology rely on cross-reactivity against *C. albicans* (Bacher et al., 2019), and other studies also showed that the host’s defense against *Klebsiella pneumoniae* (*Kp*) is up to Th17 cells and IL-17R signaling (Amezcua Vesely et al., 2019; Nakamoto et al., 2019). Both *C. albicans* and *K. pneumoniae* infections are known risk factors for ankylosing spondylitis (AS) (Li et al., 2019). Therefore, we assume that in AS, mycobiota may also synergistically deteriorate the Th17 pathology. Ankylosing spondylitis is the prototype of inflammatory rheumatic diseases grouped under the term spondyloarthritis (SpA) and represents the end phenotype of the axial SpA group (Braun and Sieper, 2007; Sieper and Poddubnyy, 2017). The pathogenesis of AS is not completely understood but likely involves a complex interplay between genetic predisposition involving the human leukocyte antigen, namely HLA-B27, and environmental factors such as mechanical stress and the microbiome (Braun and Sieper, 2007; Sieper and Poddubnyy, 2017). Up to 10% of AS patients are reported to have inflammatory bowel disease (IBD), and 70% show signs indicative of subclinical intestinal inflammation (Simone et al., 2018; Watad et al., 2018; Fragoulis et al., 2019). From genome-wide association studies, it is evident that over 10% of the gene pathways are shared between IBD and AS (Jostins et al., 2012). A recent study showed a clear link between the severity of subclinical gut inflammation and MRI-determined sacroiliac joint involvement (Van Praet et al., 2014). It has long been suggested that intrinsic barrier dysfunction permits non-specific innate immune activation with systemic translocation of bacterial adjuvants (McGonagle et al., 2001). All these factors indicate that an ecological imbalance of microbiota is a dominant risk factor in AS. In AS, the tight junctions between intestinal epithelial cells are prone to increased permeability, leading to a leaky gut (Reid et al., 2011; Koning et al., 2015). Several molecules increased in AS may point toward the idea of a leaky gut, such as lipopolysaccharide (LPS)-binding protein, fatty acid-binding protein, and zonulin (Ciccia et al., 2016; Scher et al., 2016; Ciccia et al., 2017). In recent times, specific alterations have been proposed in the composition of the gut microbiota, rather than the non-specific innate activation theory, and have been associated with a range of immune-mediated disorders (Scher et al., 2016). It has been postulated that carrying HLA-B27 is a predisposing factor for gut dysbiosis, followed by a leaky gut and the subsequent systemic entrance of microbial antigens and adjuvants, which may act as a trigger for enthesitis (Gill et al., 2018). These adjuvants may activate entheseal stromal and resident immune cell populations, leading to the activation of the IL-23/IL-17 axis and secretion of pro-inflammatory cytokines—resulting in enthesitis, osteitis, and local joint inflammation (Costello et al., 2013; Babaie et al., 2018).

In the last 5 years, more than 100 published papers have focused on the interaction between the microbiome and AS (Costello et al., 2013; Xu et al., 2016; Simone et al., 2018; Watad et al., 2018; Huang et al., 2020; Zhou et al., 2020). *Bacteroides coprophilus*, *Parabacteroides distasonis*, *Eubacterium siraeum*, *Acidaminococcus fermentans*, and *Prevotella copri* were enriched in AS patients, and pathway analysis revealed increased oxidative phosphorylation, LPS biosynthesis, and glycosaminoglycan degradation (Zhou et al., 2020). Patients with AS have a distinct fecal bacterial signature, which is linked to fecal calprotectin levels, a marker of intestinal inflammation (Klingberg et al., 2019), suggesting a local interplay between intestinal bacteria and gut inflammation in AS. While most of the previous studies were focused on the role of bacteria, few studies have focused on mycobiota, a prototypic and best studied subtype that showed that the gut mycobiota of AS patients was characterized by higher levels of Ascomycota (especially the Dothideomycetes class) and decreased abundance of Basidiomycota, mainly contributed by the death of *Agaricales*. Compared to healthy controls, AS patients showed decreased ITS2/16S biodiversity ratios and altered bacterial–fungal interkingdom networks (Li et al., 2019). Compared with nonsteroidal anti-inflammatory drugs, treatment with biological agents induced obvious changes in the gut mycobiota of AS patients, and this result was highly associated with disease activity indices, including the AS disease activity index, C-reactive protein, erythrocyte sedimentation rate, and Bath AS disease activity index (Li et al., 2019). In addition, altered mycobiota in AS patients was also found to be associated with the degree of radiographic damage (Li et al., 2019), and in the same study identified a disease-specific interkingdom network alteration in AS, suggesting that fungi, or the interkingdom interactions between bacteria and fungi, may play an essential role in AS development. In our laboratory monkey farm management, we found that some of the *Cynomolgus macaques* had similar clinical symptoms as AS patients, based on the imageological diagnosis and the clinician’s diagnosis. Therefore, in this study, we aimed to further explore the roles and interactions between bacteria and fungi in AS to prove more prototypic and best-studied subtypes of gut mycobiota and microbiota for diagnosis and treatment.

## 2 Results

### 2.1 Altered mycobiota in AS monkeys

**Figure 1:**
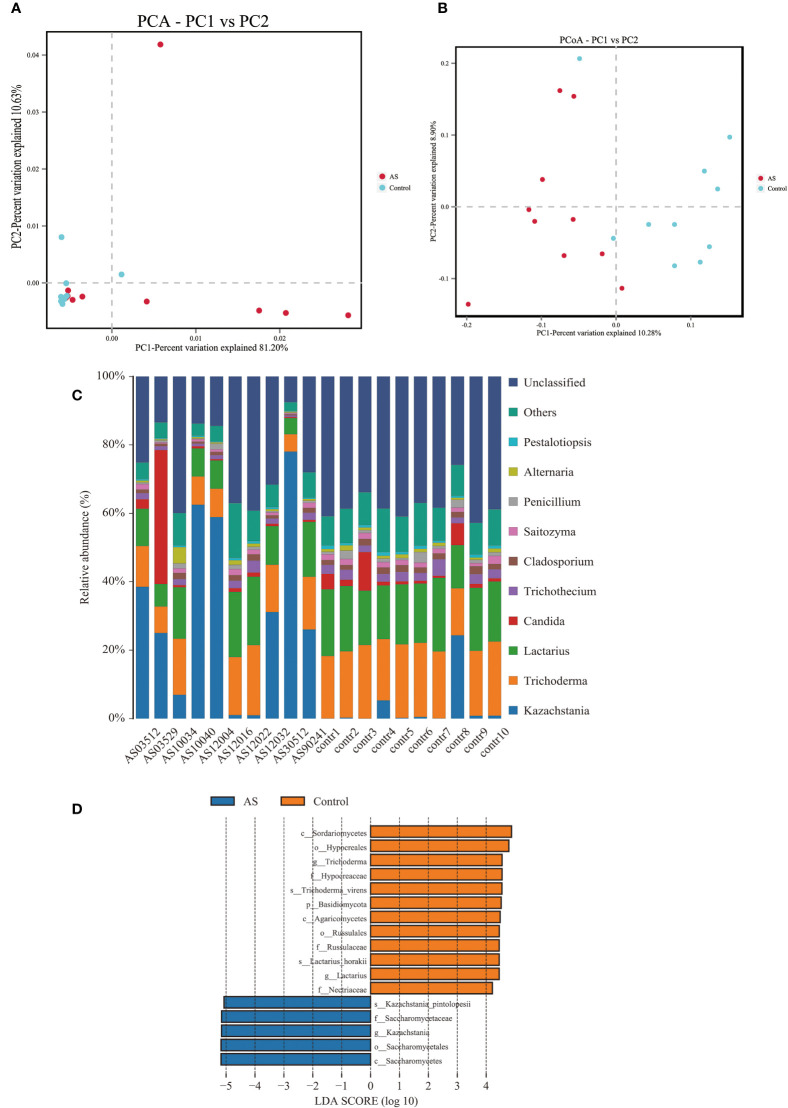
Altered mycobiota biodiversity and composition in AS monkeys. **(A)** Principal Component Analysis (PCA). Note: Dot represents each sample. Different colors represent different groups. The abscissa represents the first principal component, and the percentage represents the contribution value of the first principal component to the sample difference. The ordinate represents the second principal component, and the percentage represents the contribution of the second principal component to the sample difference. **(B)** Principal-coordinate analysis (PCoA) using the binary Jaccard distance algorithm. The horizontal and vertical coordinates are the two characteristic values that lead to the largest difference between samples, reflecting the major influence degree in the form of a percentage. **(C)** Global composition of fungal microbiota at the genus levels showing in bar chart. The more details see also in **Figures S1**, **S2**. **(D)** LEfSe (Line Discriminant Analysis (LDA) Effect Size) of AS and health monkeys. Note: The figure shows species whose LDA Score is greater than the set value (the default setting is 4.0). The length of the bar chart represents the size of the impact of different species (i.e., LDA Score). Different colors represent species in different groups.

Fecal samples from 10 AS monkeys with clinical and radiographic assessments (Jia et al.,2022) and 10 healthy controls were collected to systematically characterize thegut mycobiota and microbiota in AS monkeys by 16S rRNA and ITS2 DNAsequencing.. By sequencing ITS2, alpha diversity index difference analysis showed ACE, Chao1, Simpson, and Shannon were significant differences (**Table 1**, *p<*0.05), which indicated that the richness and diversity of mycobiota in AS were different from that of the healthy controls. The beta diversity of principal component analysis (PCA) and principal coordinate analysis (PCoA) could successfully distinguish between the two groups (**Figures 1A, B**). The histogram in **Figure 1C** showed that the mycobiota structure at the genus level in AS was different from that in the control group, and the heatmap at the genus level (**Figure S1A**) showed that most of the relative abundance of mycobiota was decreased in the AS, while a few of *Kazachstania* and *Acaulium* were increased (**Table 2**, *p<*0.05). The result of ANOVA at species level of mycobiota was shown in **Table 3**. More details of mycobiota structure are presented in **Figure S2**, which shows that AS and healthy controls could be successfully differentiated at all fungi levels, as the phyla of Rozellomycota, Basidiomycota, Mortierellomycota, Olpidiomycota, Mucoromycota, and Ascomycota (**Figure S2A**); the class of Saccharomycetes, Agaricomycetes, Archaeorhizomycetes, Sordariomycetes, Orbiliomycetes, Geminibasidiomycetes, Umbelopsidomycetes, Ustilaginomycetes, *Rozellomycotina cls Incertae sedis*, Malasseziomycetes, and Dothideomycetes (**Figure S2B**); the order of Helotiales, Thelephorales, Saccharomycetales, Cystofilobasidiales, Polyporales, Filobasidiales, and Dothideales (**Figure S2C**); the family of *Saccharomycetaceae*, *Mrakiaceae*, *Nectriaceae*, *Debaryomycetaceae*, *Filobasidiaceae*, *Ophiocordycipitaceae*, *Thelephoraceae*, and *Pichiaceae* (**Figure S2D**); the genera of *Itersonilia*, *Kazachstania*, *Trichothecium*, *Plectosphaerella*, *Pestalotiopsis*, *Apiotrichum*, *Saccharomyces*, *Ophiocordyceps*, *Pichia*, *Candida*, and *Naganishia* (**Figure S2E**); the species of *Itersonilia perplexans*, *Kazachstania pintolopesii*, *K. heterogenica*, *C. albicans*, *Nawawia malaysiana*, *Verticillium dahliae*, *Curvularia americana*, *Saccharomyces cerevisiae*, *Pichia kluyveri*, *Apiotrichum montevideense*, *Penicillium herquei*, *Ganoderma sichuanense*, *Ophiocordyceps sinensis*, and *Naganishia albida* (**Figure S2F**). The LEfSe (line discriminant analysis [LDA] effect size) results show that the *K. pintolopesii*, Saccharomycetaceae, *Kazachstania*, Saccharomycetales, and Saccharomycetes were specially enriched in the AS group (**Figure 1D**; more details regarding phylum, class, order, family, genus, and species are given in **Figure S2**).

**Table 1:** Alpha diversity index analysis of fecal mycobiota of *Cynomolgus macaques* ( $\bar{X}$ SEM).

Group	ACE	Chao1	Simpson	Shannon
Control	484.97 ± 10.8104	488.5655 ± 8.6173	0.9124 ± 0.0027	5.1997 ± 0.0578
Model	429.1262 ± 10.2794^##^	456.7695 ± 12.0992^#^	0.7747 ± 0.0568^#^	4.0852 ± 0.3825^##^

^#^p<0.05, ^##^p<0.01.

**Table 2:** ANOVA (Analysis of Variance) at genus level of mycobiota of *Cynomolgus macaques* (*p* <0.05).

Genus	Control (Mean)	Control (Sd)	AS (Mean)	AS (Sd)	Multigroup (p)
*Kazachstania*	0.032323	0.075795	0.329036	0.267534	0.003378
*Acaulium*	0.000015	0.000049	0.000138	0.000171	0.043185
*Moesziomyces*	0.000103	0.00009	0.000032	0.000038	0.033908
*Trichocladium*	0.000139	0.000183	0.000006	0.000015	0.034870
*Periconia*	0.000151	0.000158	0.000002	0.000006	0.008032
*Endophoma*	0.000279	0.00024	0.000089	0.00011	0.035337
*Chalara*	0.000443	0.000234	0.000218	0.000198	0.031681
*Botrytis*	0.000415	0.000329	0.000136	0.000235	0.04270
*Hyphozyma*	0.00049	0.000404	0.000183	0.000142	0.035568
*Subulicystidium*	0.000426	0.000434	0.000099	0.000214	0.046616
*Pseudocercospora*	0.000451	0.00047	0.000084	0.000123	0.027999
*Tomentella*	0.000814	0.000416	0.000408	0.000242	0.015801
*Oidiodendron*	0.000669	0.000464	0.000245	0.000299	0.025974
*Lecanicillium*	0.000926	0.000758	0.000368	0.000326	0.046361
*Ganoderma*	0.000943	0.000692	0.000232	0.000239	0.006558
*Trametes*	0.000813	0.000603	0.000044	0.000098	0.000879
*Saccharomyces*	0.001976	0.000586	0.001102	0.0009	0.019148
*Pichia*	0.001872	0.000933	0.000971	0.000673	0.023529
*Lasiodiplodia*	0.004023	0.001006	0.002487	0.001445	0.012912
*Plectosphaerella*	0.003823	0.001617	0.002244	0.001173	0.022295
*Naganishia*	0.002007	0.00242	0.000166	0.000263	0.027880
*Thermomyces*	0.004942	0.002004	0.003027	0.001299	0.020716
*Cladophialophora*	0.004647	0.00149	0.002615	0.001653	0.009822
*Talaromyces*	0.006753	0.003483	0.003411	0.001894	0.015743
*Pestalotiopsis*	0.007785	0.001718	0.004187	0.001765	0.000214
*Saitozyma*	0.015483	0.0038	0.010338	0.005236	0.021637
*Cladosporium*	0.0174	0.002931	0.011297	0.005201	0.004617
*Trichothecium*	0.02681	0.008617	0.016369	0.008524	0.013924
*Lactarius*	0.174983	0.024265	0.119987	0.052956	0.007930
*Trichoderma*	0.194264	0.024683	0.124402	0.049792	0.000887
Unclassified	0.375878	0.047772	0.250265	0.120199	0.006583

**Table 3:** ANOVA (Analysis of Variance) at species level of mycobiota (*p* <0.05).

Species	Control (Mean)	Control (Sd)	AS (Mean)	AS (Sd)	Multigroup (p)
*Naganishia albida*	0.001807	0.002517	0.000045	0.000077	0.040097
*Hyphozyma roseonigra*	0.00049	0.000404	0.000183	0.000142	0.035568
*Endophoma elongata*	0.000279	0.00024	0.000089	0.00011	0.035337
*Trichocladium opacum*	0.000139	0.000183	0.000006	0.000015	0.034870
*Moesziomyces aphidis*	0.000103	0.00009	0.000032	0.000038	0.033908
*Candida tropicalis*	0.000016	0.000034	0.000114	0.00013	0.032864
*Kazachstania pintolopesii*	0.026672	0.075967	0.267664	0.296121	0.022643
*Mortierella alpina*	0.001909	0.000677	0.001151	0.000661	0.020735
*Thermomyces lanuginosus*	0.004942	0.002004	0.003027	0.001299	0.020716
*Saitozyma podzolica*	0.015021	0.003819	0.009912	0.005016	0.019561
*Saccharomyces cerevisiae*	0.001976	0.000586	0.001102	0.0009	0.019148
*Trichothecium roseum*	0.02681	0.008617	0.016369	0.008524	0.013924
*Periconia byssoides*	0.000151	0.000158	0.000002	0.000006	0.008032
*Lactarius horakii*	0.174948	0.024264	0.119896	0.052838	0.007783
*Pichia kluyveri*	0.001651	0.000803	0.000753	0.00049	0.007335
*Unclassified*	0.463492	0.050731	0.310721	0.148861	0.006571
*Ganoderma sichuanense*	0.000943	0.000692	0.000232	0.000239	0.006558
*Trichoderma virens*	0.191468	0.024364	0.122351	0.049409	0.000903
*Trametes versicolor*	0.000435	0.000273	0.000035	0.00007	0.00028
*Penicillium herquei*	0.001819	0.000913	0.000445	0.000269	0.000240

### 2.2 Altered bacterial microbiota in AS monkeys

**Figure 2:**
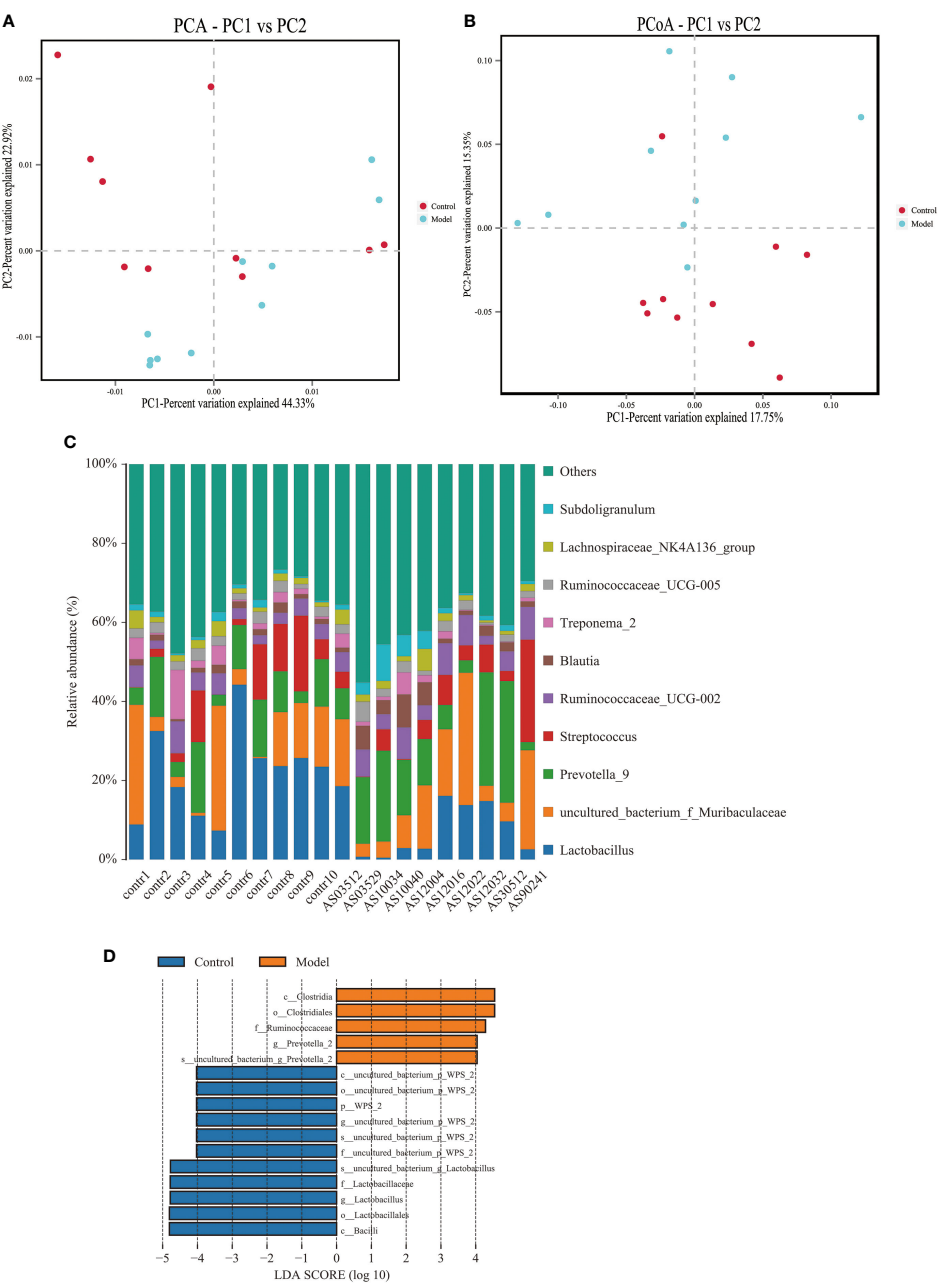
Altered bacterial microbiota biodiversity and composition in AS monkeys. **(A)** Principal Component Analysis (PCA). Note: Dot represents each sample. Different colors represent different groups. The abscissa represents the first principal component, and the percentage represents the contribution value of the first principal component to the sample difference. The ordinate represents the second principal component, and the percentage represents the contribution of the second principal component to the sample difference. **(B)** Principal-coordinate analysis (PCoA) using the binary Jaccard distance algorithm. The horizontal and vertical coordinates are the two characteristic values that lead to the largest difference between samples, reflecting the major influence degree in the form of a percentage. **(C)** Global composition of bacterial microbiota at the genus levels showing in bar chart. The more details see also in **Figures S1**, **S2**. **(D)** LEfSe (Line Discriminant Analysis (LDA) Effect Size) of AS and health monkeys. Note: The figure shows species whose LDA Score is greater than the set value (the default setting is 4.0). The length of the bar chart represents the size of the impact of different species (i.e., LDA Score). Different colors represent species in different groups.

We analyzed the bacterial fraction of the microbiota using high-throughput sequencing of the bacterial 16S rRNA gene. Alpha diversity index difference analysis showed only ACE was significant differences (**Table 4**, *p<*0.05), which indicated that the species abundance in normal group was higher than that in AS group, but the diversity of bacterial microbiota was no different between two groups. The beta diversity of principal component analysis (PCA) and principal coordinate analysis (PCoA) could successfully distinguish between the two groups (**Figures 2A, B**). The histogram in **Figure 2C** showed that the bacterial microbiota structure at the genus level in AS was different from the control group, and the heatmap at the genus level (**Figure S3A**) showed that *Prevotella 2*, *Blautia*, *Faecalibacterium*, *Lachnospiraceae UCG-008*, an *uncultured bacterium of Erysipelotrichaceae*, *Ruminococcaceae UCG-003*, an *uncultured bacterium of Lachnospiraceae*, *Negativibacillus* and *Mogibacterium* were increased, while the others were decreased (**Table 5**, *p<*0.05). The result of ANOVA at species level of bacterial microbiota of Cynomolgus macaques was shown in **Table 6**. More details with respect to phylum, class, order, family, genus, and species are shown in **Figure S4**. A LefSe analysis was adopted to identify the bacterial groups that showed considerable and significant differences between the AS and control groups. As shown in **Figure 2D**, the comparison between the AS and control groups revealed that *Prevotalla 2*, an *uncultured bacterium of Prevotella 2*, *f Ruminococcaceae*, *Clostridia*, and *Clostridiales* were specially enriched in the AS group.

**Table 4:** Alpha diversity index analysis of fecal bacterial microbiota of Cynomolgus macaques ( $\bar{X}$ ± SEM).

Group	ACE	Chao1	Simpson	Shannon
Control	508.5486 ± 5.6897	511.805 ± 5.822	0.9282 ± 0.0098	5.7056 ± 0.118
Model	490.5584 ± 5.2798^#^	495.4398 ± 6.3334	0.9436 ± 0.0125	5.9536 ± 0.1826

^#^p<0.05.

**Table 5:** ANOVA (Analysis of Variance) at genus level of bacterial microbiota of Cynomolgus macaques (*p* <0.05).

Genus	Control (Mean)	Control (Sd)	Model (Mean)	Model (Sd)	Multigroup (p)
*Prevotella 2*	0.009071	0.002864	0.030089	0.019495	0.003388
*Blautia*	0.015087	0.005699	0.033201	0.025564	0.042185
*Faecalibacterium*	0.006059	0.00231	0.019842	0.01799	0.02726
*Lachnospiraceae UCG-008*	0.009898	0.003865	0.015706	0.006952	0.033016
*Erysipelotrichaceae*	0.001115	0.000652	0.004646	0.004907	0.036807
*Ruminococcaceae UCG-003*	0.002625	0.002321	0.004968	0.002626	0.048714
*Lachnospiraceae*	0.000982	0.000331	0.002676	0.001743	0.007393
*Negativibacillus*	0.000652	0.00027	0.001853	0.001133	0.004341
*Mogibacterium*	0.000726	0.000444	0.001356	0.000768	0.037399
*Catenibacterium*	0.00035	0.000121	0.000949	0.000692	0.01472
*Libanicoccus*	0.000174	0.000132	0.000372	0.000236	0.032824
*Sphingomonadaceae*	0.000177	0.00015	0.000072	0.000038	0.044976
*Gemmatimonadaceae*	0.00014	0.00011	0.000032	0.000028	0.007292
*CAG-352*	0.000266	0.000149	0.000108	0.000057	0.005916
*RB41*	0.000166	0.000156	0.000002	0.000007	0.003701
*Ruminococcus 2*	0.000727	0.000486	0.000345	0.000266	0.042954
*Ruminococcaceae NK4A214 group*	0.009494	0.00389	0.005877	0.003794	0.049609
*p_WPS-2*	0.022002	0.031256	0.000117	0.000148	0.039959
*Lactobacillus*	0.22074	0.113329	0.08226	0.070856	0.004194

**Table 6:** ANOVA (Analysis of Variance) at species level of bacterial microbiota of Cynomolgus macaques (*p* <0.05).

Species	Control(Mean)	Control(Sd)	Model(Mean)	Model(Sd)	Multigroup(p)
*g_Prevotella_2*	0.009071	0.002864	0.030089	0.019495	0.003388
*g_Blautia*	0.015087	0.005699	0.033201	0.025564	0.042185
*g_Faecalibacterium*	0.006059	0.00231	0.019842	0.01799	0.02726
*g_Lachnospiraceae_UCG-008*	0.009898	0.003865	0.015706	0.006952	0.033016
*f_Erysipelotrichaceae*	0.001115	0.000652	0.004646	0.004907	0.036807
*g_Ruminococcaceae_UCG-003*	0.002625	0.002321	0.004968	0.002626	0.048714
*f_Lachnospiraceae*	0.000982	0.000331	0.002676	0.001743	0.007393
*g_Negativibacillus*	0.000652	0.00027	0.001853	0.001133	0.004341
*g_Mogibacterium*	0.000726	0.000444	0.001356	0.000768	0.037399
*g_Catenibacterium*	0.00035	0.000121	0.000949	0.000692	0.01472
*g_Libanicoccus*	0.000174	0.000132	0.000372	0.000236	0.032824
*f_Sphingomonadaceae*	0.000177	0.00015	0.000072	0.000038	0.044976
*f_Gemmatimonadaceae*	0.00014	0.00011	0.000032	0.000028	0.007292
*g_CAG-352*	0.000266	0.000149	0.000108	0.000057	0.005916
*g_RB41*	0.000166	0.000156	0.000002	0.000007	0.003701
*g_Ruminococcus_2*	0.000727	0.000486	0.000345	0.000266	0.042954
*Streptococcus_troglodytidis*	0.000535	0.000729	0.000044	0.000051	0.047559
*g_Ruminococcaceae_NK4A214_group*	0.009494	0.00389	0.005877	0.003794	0.049609
*bacterium_p_WPS-2*	0.022002	0.031256	0.000117	0.000148	0.039959
*g_Lactobacillus*	0.216994	0.111853	0.080477	0.068877	0.004102

### 2.3 AS monkeys showed altered bacterial-fungal associations

**Figure 3:**
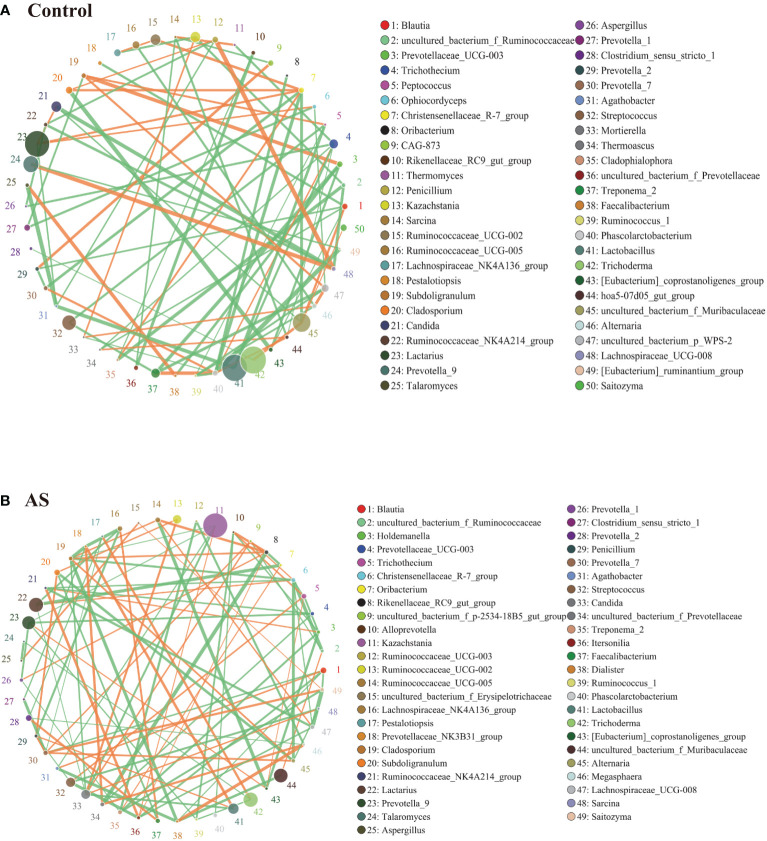
Bacterial–fungal association analysis in the AS and health monkeys. Note: Network graph is a form of correlation analysis. A Spearman rank correlation analysis was conducted according to the abundance and change of each species in each sample, and the data with correlations greater than 0.1 and *P<*0.05 were screened to construct a correlation network. Based on network graph analysis, the coexistence relationship of species in environmental samples can be obtained, as can the interaction situation and important pattern information of species in the same environment, and the formation mechanism of phenotypic differences between samples can be further explained. **(A)** Correlation analysis of fecal microorganisms (fungi and bacteria) in healthy monkeys. **(B)** Correlation analysis of fecal microorganisms (fungi and bacteria) in AS monkeys. Note: The circle represents species, and the size of the circle represents the average abundance of species. The lines represent the correlation between two species, the thickness of the lines represents the strength of the correlation, and the color of the lines: orange represents a positive correlation and green represents a negative correlation.

Fungi and bacteria coexist in the human gut and interact with each other. Although they have been shown to contribute actively to health or disease, few studies have investigated whether the fungal microbiota in AS patients is perturbed. The alpha diversity indexes of Chao1, Ace, Shannon, and Simpson were significantly different (**Table 1**, *p<*0.05), which indicated that the richness and diversity of fungi and bacteria in AS were different from the healthy controls. The beta diversity of PCA and PCoA could successfully distinguish between both groups (**Figure 1A**). In addition to composition differences, we found that the microbiota network (which includes bacteria and fungi) at the genus level in AS monkeys was notably different from that in healthy controls (**Figure 3**). Specifically, the density of the network in AS monkeys was remarkably higher than that of the healthy individuals (**Figure 3**, *r >*0.1, *p<*0.05, top 50), which suggested an alteration of the entire ecosystem in the guts of the AS monkeys. A higher Spearman’s correlation was found in AS monkeys (104 correlation) compared with healthy controls (90 correlation) (**Figure 3**). According to the report of Li et al. on AS patients in northeast China, these networks were different (Li et al., 2019), which may be due to the limiting bacterial but not fungal environment of laboratory monkeys or the high temperature, humidity, and climatic conditions in southern China. However, these results need further epidemiological validation. Interestingly, in AS monkeys, we observed a positive correlation between the abundance of *Candida* and *Ruminococcaceae UCG-002*, *Sarcina*, and the *p-2534-18B5 gut* family group, and a negative correlation with *Cladosporium*. *Blautia* showed a negative correlation with *Dialister*, *Itersonilia*, *Prevotella 7*, *Saitozyma*, and a positive correlation with *Agathobacter.* The *Christensenellaceae R-7 group* showed a negative correlation with *Alternaria*, *Cladosporium*, *Lachnospiraceae UCG-008*, *Ruminococcaceae NK4A214 group*, and uncultured bacterium *Ruminococcaceae*. *Kazachstania* showed a negative correlation with *Agathobacter*, *Cladosporium*, *Prevotella* 9, and *Candida* and a positive correlation with *Itersonilia*. Lachnospiraceae UCG-008 showed a negative correlation with *Itersonilia*, the *Ruminococcaceae NK4A214 group*, and *Saitozyma* and a positive correlation with the uncultured bacterium Erysipelotrichaceae. *Prevotella 7* showed a negative correlation with *Clostridium sensu stricto* 1 and *Pestalotiopsis* and a positive correlation with *Alternaria*, *Penicillium*, and *Saitozyma*. *Treponema* 2 showed a negative correlation with *Holdemanella*, *Prevotella 1*, and *Saitozyma*. *Lactobacillus* showed a negative correlation with *Candida* and the *Prevotellaceae NK3B31 group* and a positive correlation with *Penicillium*. *Megasphaera* showed a positive correlation with *Dialister* and a negative correlation with *Prevotella* 2 and *Ruminococcaceae UCG-003*. More details are given in **Figure 3** (*r >*0.1, *p<*0.05, top 50). Taken together, these results suggest a complex relationship between bacteria and fungi in the gut microbiota. Further studies are needed to better understand the specific communication and interaction between the host and its resident bacteria.

### 2.4 *K. pintolopesii* induces multiple AS immune activation phenotypes in mice

**Figure 4:**
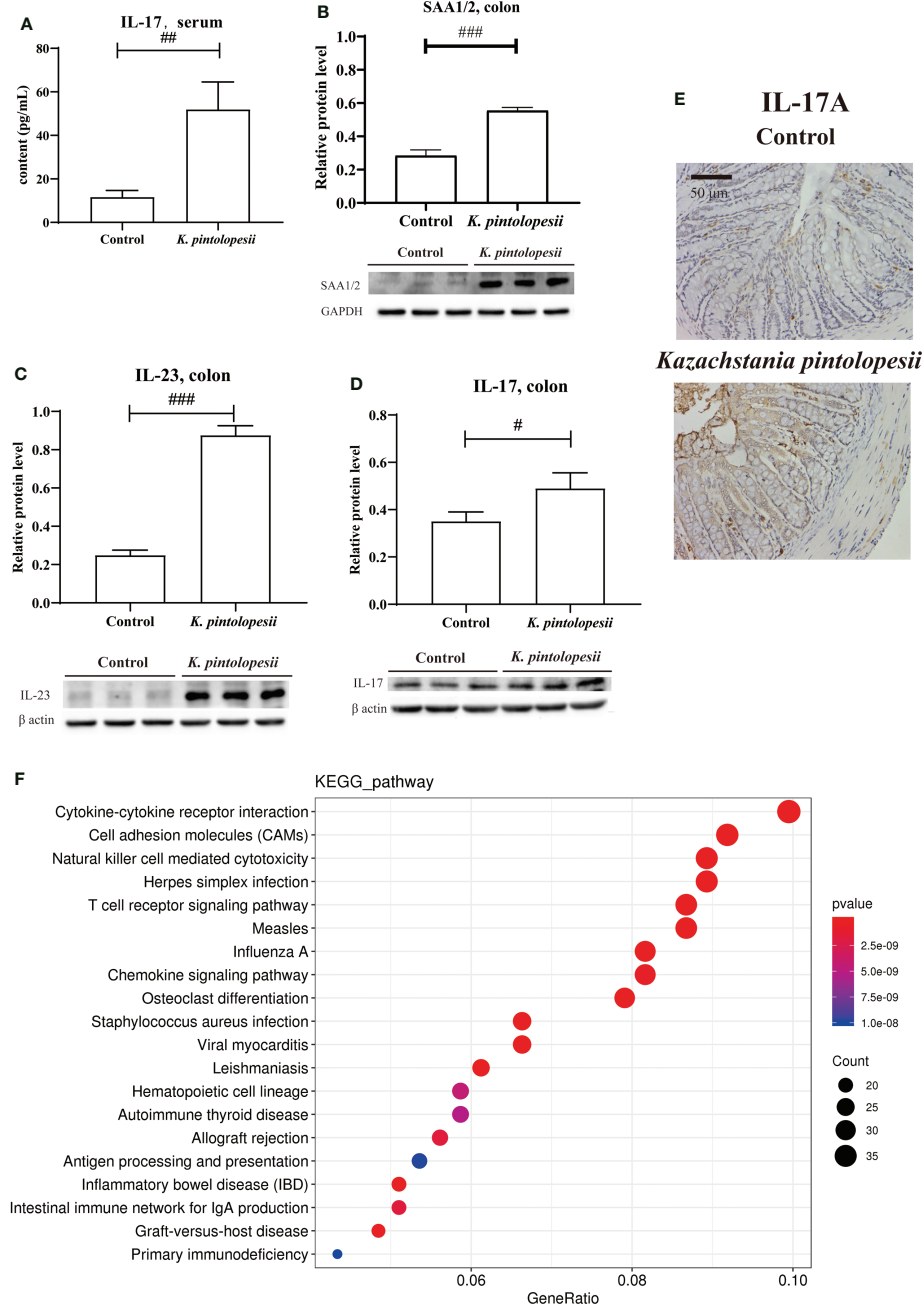
*K pintolopesii* induces multiple AS immune activation phenotypes in mice. **(A)** *K pintolopesii* upregulates the levels of IL-17 in serum (*p<*0.05). **(B)** *K pintolopesii* upregulates the expression of SAA1/2 in colon tissues (*p<*0.05). **(C)** *K pintolopesii* upregulates the expression of IL-23 in colon tissues (*p<*0.05). **(D)** *K pintolopesii* upregulates the expression of IL-17 in colon tissues (*p<*0.05). **(E)** The expression of IL17A in colon tissue was detected by immunohistochemistry. **(F)** KEGG analysis of differentially expressed genes showed that the pathways of *cytokine–cytokine receptor interaction*, *cell adhesion molecules* (CAMs), *natural killer cell-mediated cytotoxicity*, *Herpes simplex infection*, *T-cell receptor signaling pathway*, *measles*, *influenza A*, *chemokine signaling pathway*, *osteoclast differentiation*, *autoimmune thyroid disease*, and *inflammatory bowel disease* (IBD) were enriched pathways (*p<*0.05). ^#^p < 0.05, ^##^p < 0.01, ^###^p < 0.001.

*K. pintolopesii* was purchased from CBS strains in the Netherlands under platform number Bio-109890. For more information, visit http://www.biobw.org/International-strain/bio-109890.html. Then, *K. pintolopesii* (7.0 × 10^8^ CFU/ml) was administered to mice by intragastrical for the last 7 days. Our results showed that the serum levels of IL-17A were significantly upregulated (**Figure 4A**, *p<*0.05), and the immunohistochemical staining of the colon showed that *K. pintolopesii* could obviously upregulate the expression of the IL-17A protein (**Figure 4E**). Further, the expression of SAA1/2, IL-23, and IL-17 were significantly upregulated (**Figures 4B–D**, *p<*0.05). The RNA sequencing of colonic tissues showed that about 866 mRNAs should have a change in expression, with 211 mRNAs upregulated and 655 downregulated (fold change [FC] ≥1.5 and FDR<0.05). The KEGG analysis of DEGs showed that the pathways of *Cytokine–cytokine receptor interaction*, *Cell adhesion molecules* (CAMs), *Natural killer cell-mediated cytotoxicity*, *Herpes simplex infection*, *T-cell receptor signaling pathway*, *Measles*, *Influenza A*, *Osteoclast differentiation*, *Staphylococcus aureus infection*, *Viral myocarditis*, *Hematopoietic cell lineage*, *Autoimmune thyroid disease*, *Allograft rejection*, *Antigen processing and presentation*, *Inflammatory bowel disease* (IBD), *Intestinal immune network for IgA production*, *Graft-versus-host disease*, and *Primary immunodeficiency* (**Figure 4F**) were the top enriched pathways. The GO classification of DEGs showed that the biological processes of *immune system*, *inflammatory response* (**Figure S5A**, *p<*0.05); cellular components of *extracellular exosomes* and cytosol (**Figure S5B**, *p<*0.05); and molecular functions of *antigen binding*, *immunoglobulin receptor binding*, *MHC class Ⅱ protein binding*, and *CD8 receptor binding* were enriched (**Figure S5C**, *p<*0.05). Furthermore, most pathways were related to infection immunity and inflammation.

Deep analysis showed that the mRNAs of *Gzma* (*p* = 1.39E−09), *Gzmb* (*p* = 1.20E−06), *Pik3r5*, *Map3k14*, *Ctsw*, *Casp12*, *Traf1*, *Prf1*, *Pik3cd*, *Jun*, *Csf2rb*, *Csf2rb2*, *Bcl2a1b*, and *Bcl2a1d* showed changes induced by *K. pintolopesii*. Granzyme B (GzmB) and Granzyme A (GzmA) are apoptotic proteinase-like serine proteases that can activate caspase-3 and caspase-8 and be transferred to target cells by cytotoxic lymphocytes to trigger cell apoptosis, pyroptosis, and kill virus-infected cells and tumor cells (Jiang et al., 2020; Zhang et al., 2020). This indicated that the necroptosis, pyroptosis, and/or apoptosis delay of neutrophils, lymphocytes, natural killer cells, and/or CD8 cytotoxic T killer cells were triggered by *K. pintolopesii*, which eventually induces autoimmune diseases or other inflammatory diseases. The changes of *Il12rb1*, *Cd40*, *Il12rb2*, *Cxcl16*, *Ccl9*, *Ifnar2*, *Ltb*, *Csf1r*, *Il18rap*, *Il18r1*, *Il2ra*, *Bmp2*, *Il1b*, *Pdgfc*, *Csf3r*, *Cxcl9*, *Ccr9*, *Cd27*, *Il21r*, *Il4ra*, *Il2rg*, *Pdgfd*, *Il10ra*, *Crlf2*, *Ccl5*, *Ccl2*, *Inhbb*, *Il18*, *Cxcr6*, *Ccr2*, *Cxcr3*, *Cx3cr1*, *Cxcl2*, *Xcr1*, *Il2rb*, *Csf2rb*, *Csf2rb2*, *Ccl28*, and *Ccr5* mRNAs were enriched into the *cytokine–cytokine receptor interaction*; and previous studies have demonstrated that the chemokine receptor gene *CXCR6* was highly expressed in +CPI colitis T cells and a variety of human cancer cells (Luoma et al., 2020). However, *CXCL16*, a CXCR6 ligand gene regulated by IFN and TNF signaling pathways, also showed significantly high expression in bone marrow cells and mast cells (Xiao et al., 2015), suggesting that CXCR6 inhibition could be a potential therapeutic target to reduce the metastasis of cancer cells and effectively improve intestinal inflammation. Thus, *K. pintolopesii* may be a trigger for the AS complication of intestinal inflammation. The changes of *Itgb2*, *Lck*, *Lcp2*, *Ptpn6*, *Cd244a*, *Cd247*, *Vav2*, *Cd48*, *Gzmb*, *Fyn*, *Pik3r5*, *Ppp3cc*, *Ifnar2*, *Zap70*, *Klrk1*, *Klrd1*, *Klrc1*, *Tyrobp*, *Lat*, *Itgal*, *Nfatc1*, *Rac2*, *Vav3*, *Prf1*, *Icam1*, *Pik3cd*, *Mus_newGene_6834*, *Fcgr4*, *Prkcb*, *Sh3bp2*, *Fcer1g*, and *Mus_newGene_6925* were enriched into the *Natural killer cell mediated cytotoxicity*; and the changes of *Itgb2*, *Cd86*, *Ltb*, *Ctla4*, *Cd28*, *Il1b*, *Itgal*, *Ccl5*, *Ccl2*, *H2-Aa*, *Icam1*, *H2-DMa*, *Il18*, *Jun*, *H2-Eb1*, *H2-Ab1*, *H2-DMb1*, *Mus_newGene_3650*, *Mus_newGene_6834*, and *Mus_newGene_6925* were enriched in *rheumatoid arthritis*, all of which indicated that *K. pintolopesii* is a trigger of AS-induced multiple AS immune activation phenotypes. Further details of other pathways are shown in **Table S1**.

Gene Set Enrichment Analysis (GSEA) using DEXSeq (version 1.12.2, default parameters) showed that *positive regulation of GTPase activity*, *innate immune response* (**Figure S6A**), *inflammatory response* (**Figure S6B**), *G-protein coupled receptor signaling pathway*, *positive regulation of gene expression*, *response to lipopolysaccharide*, *positive regulation of ERK1 and ERK2 cascade*, *regulation of cell shape*, *cellular response to lipopolysaccharide*, and *immune response* were the top 10 enriched biological processes (*p<*0.0015). The *integral component of plasma membrane* (**Figure S6C**), *external side of plasma membrane*, *membrane raft*, *transcription factor complex* (**Figure S6D**), *lamellipodium*, *cell–cell junction*, *actin filament*, *MHC class II protein complex*, *Golgi medial cisterna*, *multivesicular body*, and *immunological synapse* were the top 12 enriched cellular components (*p<*0.0016). *GTP binding*, *GTPase activity*, *carbohydrate binding* (**Figure S6E**), *antigen binding* (**Figure S6F**), *integrin binding*, *receptor activity*, *protein binding*, *peptide antigen binding*, *transmembrane signaling receptor activity*, *virus receptor activity*, *cytokine receptor activity*, and *T-cell receptor binding* were the top 12 enriched molecular functions (*p<*0.0016).

DEU analysis (DEXSeq was used to analyze DEU and performed FDR as the criterion<0.05, **Table 7**) showed that the exon E020 of *Nlrp1b*, a key mediator of programmed cell death, was changed (*p* = 2.07E−07). Overexpression of this gene was reported to induce apoptosis and pyroptosis, and this gene mediates diseases including palmoplantar carcinoma, multiple self-healing, and autoinflammation with arthritis and dyskeratosis, so *K. pintolopesii* could influence the exon E020 of *Nlrp1b* to mediate programmed cell death. The E003 (*p* = 3.55E−17) and E005 (*p* = 1.29E−15) of *Bri3* were different, and a previous study has shown that Bri3 participates in TNFα-induced cell death (Wu et al., 2003). The E001 (*p* = 5.79E−07) and E004 (*p* = 7.38E−10) of Efhd2, which were demonstrated to regulate B-cell receptor (BCR)-induced immature and primary B-cell apoptosis (Kroczek et al., 2010) and play a role as a negative regulator of the canonical NF-κB activating branch (Avramidou et al., 2007) and control spontaneous apoptosis through the regulation of BCL2L1 abundance (Avramidou et al., 2007). Furthermore, there were another 23 exons in different genes that also changed (**Table 7**, FDR<0.05). Taken together, these data show that *K. pintolopesii* could influence cell pyroptosis and induce inflammation.

**Table 7:** DEU Analysis at exon levels in *K. pintolopesii* treated mice, DEXSeq was used to analysis DEU and performed FDR as the criterion<0.05.

Gene	Exon ID	log2(FC)	*p*-value	FDR
Pam	E034	−0.3609	1.21E−10	2.92E−06
Mfsd4a	E005	−0.3961	1.18E−06	0.0071
Nus1	E001	−0.1146	1.42E−08	0.0002
Hnrnpab	E010	−0.4666	3.08E−11	8.90E−07
Hspa4	E019	−0.1479	3.51E−07	0.0028
Nlrp1b	E020	−1.5074	2.07E−07	0.0019
Casc3	E003	−0.5852	3.67E−07	0.0028
Tmem117	E002	−0.5752	1.02E−06	0.0064
Osbpl11	E011	−0.8746	8.16E−08	0.0008
Dop1b	E028	−6.5427	7.85E−07	0.0054
Dynlt1f	E003	0.2918	2.07E−07	0.0019
Prepl	E017	1.4704	2.78E−11	8.90E−07
Carnmt1	E005	0.7411	2.55E−09	4.61E−05
Fam78a	E001	2.0776	1.03E−12	4.97E−08
Rgs3	E022	−2.1000	1.56E−08	0.0002
Efhd2	E001	0.0249	5.79E−07	0.0042
Efhd2	E004	−0.1541	7.38E−10	1.53E−05
Shroom3	E012	−0.2932	5.48E−08	0.0006
Bri3	E003	−0.7228	3.55E−17	5.14E−12
Bri3	E005	0.1934	1.29E−15	9.35E−11
Pde6h	E002	−5.0913	1.37E−06	0.0079
Eif3f	E006	0.1168	9.33E−07	0.0061
Psmb10	E008	0.3635	3.61E−07	0.0028
Rpl13	E006	0.1444	1.83E−06	0.0098
Dcun1d5	E002	−0.3293	2.73E−08	0.0003
Cnn1	E002	0.6703	1.83E−06	0.0098
Rpsa	E004	0.0758	1.06E−08	0.0002

gene ID, gene identifier; exon ID, exon number; Log2(FC), the fold change normalized by log2 of expression; p-value, p-value for significance of differential exon; FDR, False Discovery Rate.

### 2.5 *K. pintolopesii* induces PANoptosis of macrophage RAW264.7

**Figure 5:**
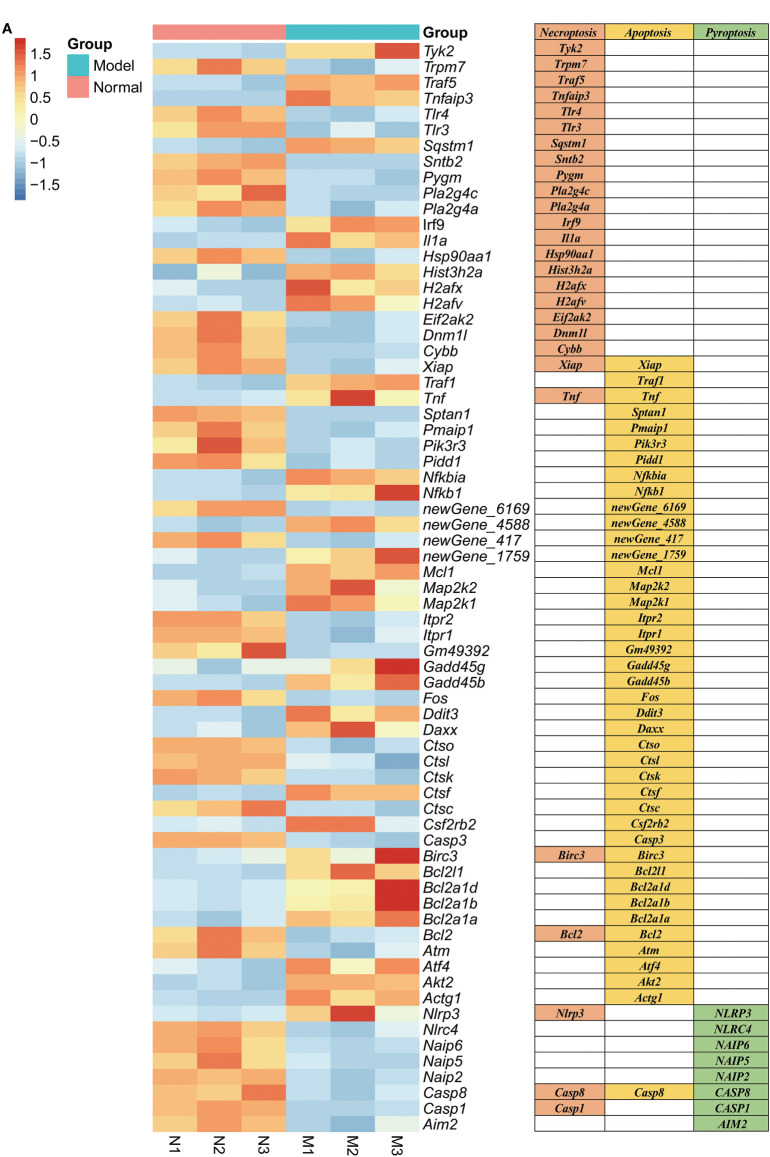
Genes related to PANoptosis were screened from differentially expressed genes in the transcriptome of RAW264.7 co-cultured with *K. pintolopesii*. **(A)** Heatmap of differentially expressed genes related to PANoptosis.

RNA sequencing of RAW264.7 co-cultured with *K. pintolopesii* showed that about 2,622 mRNAs changed in expression, with 1,016 mRNAs upregulated and 1,606 downregulated (fold change [FC] ≥1.5 and FDR<0.05, **Figure S7A**). The differentially expressed genes related to apoptosis were *Daxx*, *Akt2*, *Map2k1*, *Bcl2l1*, *Gadd45b*, *Nfkbia*, *Fos*, *Gadd45g*, *Ctsl*, *Tnf*, *Pmaip1*, *Ddit3*, *Pidd1*, *Xiap*, *Casp8*, *Traf1*, *Ctso*, *Ctsk*, *Nfkb1*, *Pik3r3*, *Itpr1*, *Itpr2*, *Ctsc*, *Casp3*, *Birc3*, *Atm*, *Map2k2*, *Mcl1*, *Atf4*, *Bcl2*, *Sptan1*, *Actg1*, *Csf2rb2*, *Ctsf*, *Bcl2a1b*, *Bcl2a1d, Bcl2a1a*, and *Gm49392* (**Figure 5A**); related to necroptosis were *Irf9*, *Cybb*, *Sqstm1*, *Tnfaip3*, *Hsp90aa1*, *Dnm1l*, *Eif2ak2*, *Tnf*, *Xiap*, *Casp1*, *Casp8*, *Traf5*, *Trpm7*, *Il1a*, *Tlr3*, *Birc3*, *Tyk2*, *Pygm*, *Nlrp3*, *Pla2g4c*, *Tlr4*, *H2afv*, *Sntb2*, *H2afx*, *Pla2g4a*, *Bcl2*, and *Hist3h2a* (**Figure 5A**); and related to pyroptosis were *NLRP3*, *NLRC4*, *NAIP6*, *NAIP5*, *NAIP2*, *CASP8*, *CASP1*, and *AIM2* (**Figure 5A**), which suggested that *K. pintolopesii* may induce PANoptosis in RAW264.7 cells.

**Figure 6:**
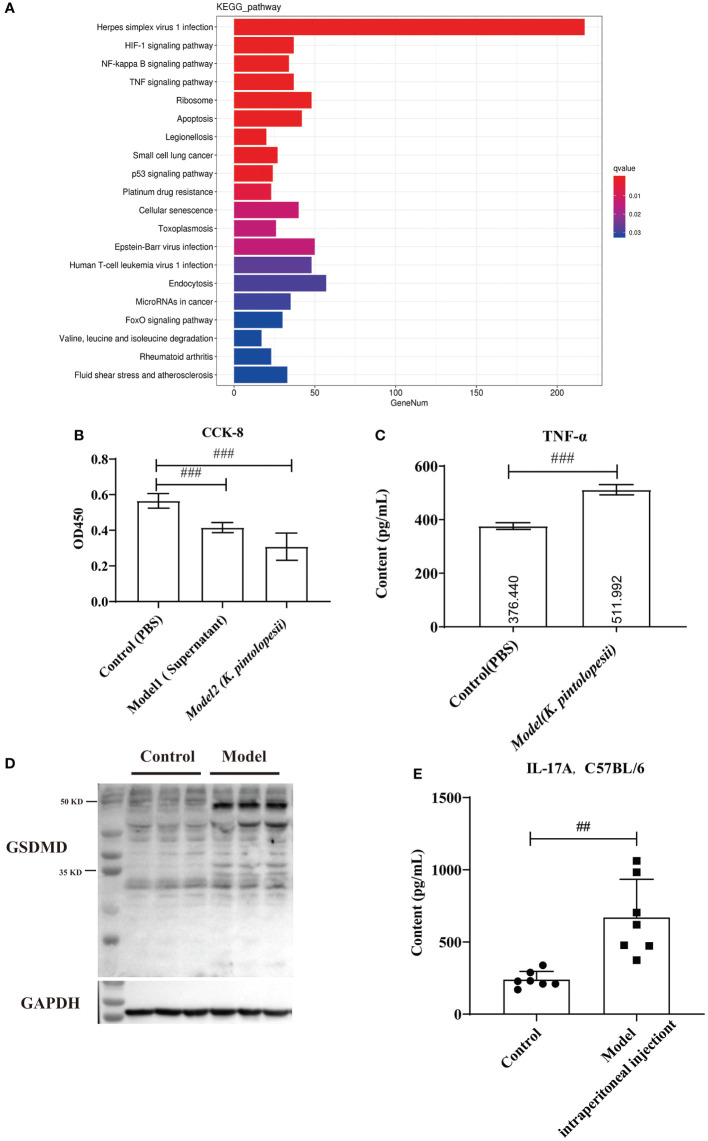
The effect of *K. pintolopesii* on macrophage RAW264.7 **(A)** KEGG analysis of transcriptome of RAW264.7 treated by *K. pintolopesii*. **(B)** CCK8 was used to detect of RAW264.7 treated by the supernatant of *K. pintolopesii* or *K. pintolopesii* **(C)** The content of TNF-α of the supernatant of *K. Pintolopesii* co-cultured RAW264.7 was determined by Elisa. **(D)** Western blot was used to detect the GSDMD protein level of RAW264.7 after co-culture with *K. pintolopesii*. **(E)** The levels of IL-17A in the serum of mice infected by the products (culture supernatant) secreted by *K. pintolopesii* through intraperitoneally were determined by Elisa. ^#^p < 0.05, ^##^p < 0.01, ^###^p < 0.001.

The KEGG analysis of DEGs showed that the pathways of *Herpes simplex virus 1 infection*, *HIF-1 signaling pathway*, *NF-kappaB signaling pathway*, *TNF-signaling pathway*, *ribosome*, *apoptosis*, *legionellosis*, *small cell lung cancer*, *p53 signaling pathway*, *platinum drug*, *cellular senescence*, *toxoplasmosis*, *Epstein–Barr virus infection*, *human T-cell leukemia virus 1 infection*, *endocytosis*, *microRNAs in cancer*, *FoxO signaling pathway*, *valine, leucine and isoleucine degradation*, *rheumatoid arthritis*, *fluid shear stress*, and *atherosclerosis* were the top enriched pathways (**Figures 6A**, **S7B, C**). The KEGG analysis did not enrich the pyroptosis-related pathways because the KEGG data did not include the cell apoptosis pathway yet. The GO classification of DEGs showed that the biological processes of regulation of *apoptotic processes*, *positive regulation of NF-kappaB transcription* (**Figure S8A**, *p<*0.05), cellular components of *nucleus*, *cytoplasm*, and *cytosol* (**Figure S8B**, *p<*0.05), and molecular functions of *peptide antigen binding* and *T-cell receptor binding* were enriched (**Figure S8C**, *p<*0.05). Furthermore, most pathways were related to the infection immunity and inflammation. There is no doubt that *K. pintolopesii* can obviously influence the function of macrophages.

In addition tests showed that the number of living cells was decreased significantly (*p<*0.05) after 6 h co-cultured with the supernatant of *K. pintolopesii* or *K. pintolopesii* using CCK8, and many floating cells (**Figure 6B**); and the content of TNF-α of the supernatant of *K. Pintolopesii* co-cultured RAW264.7 was significantly increased (**Figure 6C**, *p<*0.05), also the GSDMD protein levels (**Figure 6D**), which indicated that *K. pintolopesii* can cause the pyroptosis. Furthermore, the levels of IL-17A in the serum of mice infected by the products (culture supernatant) secreted by *K. pintolopesii* intraperitoneally were significantly higher (**Figure 6E**, *p<*0.05). This suggested that *K. pintolopesii* and their secreted products could induce severe inflammatory reactions *in vivo* and *in vitro.*

### 2.6 PANoptosis products promote the proliferation, biofilm formation, and morphological changes of *K. pintolopesii*

**Figure 7:**
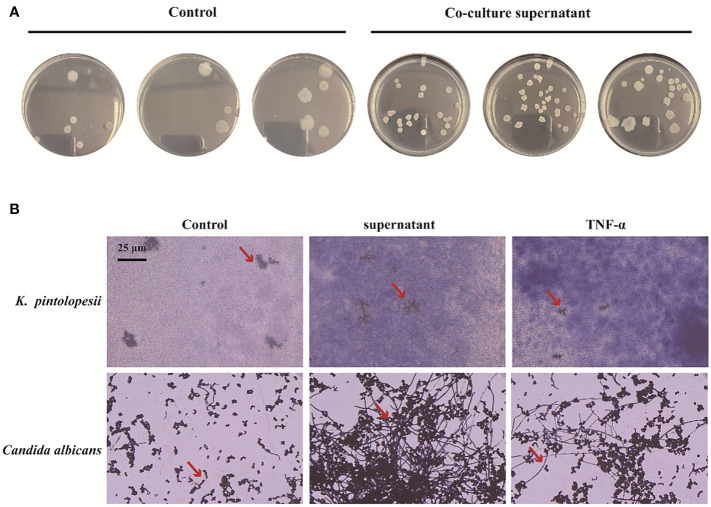
Effect of PANoptosis products on K. pintolopesii. **(A)** Plate count. PANoptosis products significantly promotes K. pintolopesii proliferation. **(B)** PANoptosis products and TNF-α (Final concentration of 1000 pg/mL) could change the morphology of *K. pintolopesii* and *Candida albicans*.

To determine the next reaction of PANoptosis induced by *K. pintolopesii*, PANoptosis products were added into the culture of *K. pintolopesii.* Unexpectedly, the colony of *K. pintolopesii* showed positive correction to the PANoptosis products (**Figure 7A**), which indicated that the PANoptosis products could promote the proliferation of *K. pintolopesii*. The morphology of *K. pintolopesii* were changed in the PANoptosis products and TNF-α (Final concentration of 1,000 pg/ml) could change (**Figure 7B**), as the same of *C. albicans* changed from the yeast state to the mycelium state. Those findings suggest that chronic inflammation may cause the transformation of conditioned pathogenic fungi from yeast to mycelium, thereby increasing their pathogenicity. The mechanism of morphological changes of opportunistic pathogens caused by host inflammatory factors is still unclear, and further experiments are needed to elucidate the mechanism. It is suggested that the exotoxin of *K. pintolopesii* (compared to *C. albicans*) may be an important substance in activating the IL-17RA pathway.

## 3 Discussion

In this study, fecal samples from 10 AS monkeys with clinical and radiographic assessments and 10 healthy controls were collected to systematically characterize the gut mycobiota and microbiota in AS by 16S rRNA gene- and ITS2-based DNA sequencing. Our results showed that the gut mycobiota of *K. pintolopesii*, *I. perplexans*, *Saccharomycetaceae*, *Coniochaeta*, and *Coniochaetaceae* were especially enriched in AS. The microbiota of AS monkeys was characterized by increased abundance of the *Prevotalla 2* genus, *Ruminococcaceae* family, Clostridia class, and Clostridiales order. Compared to healthy controls, decreased ITS2/16S biodiversity ratios and altered bacterial-fungal interkingdom networks were observed in AS monkeys. All these findings implied that fungal and bacterial dysbiosis are closely linked to AS and that their communication and interaction play an important role in autoimmune response.

In this paper, the LEfSe analysis showed that the biomarker fungi and bacteria of *Saccharomycetaceae*, *Saccharomycetes*, *Kazachstania*, and *K. pintolopesii* were rich in AS, while *Sordariomycetes*, *Lactobacillaceae*, *Lactobacillus*, *Hypocreales*, *L. horakii*, *Trichoderma*, *Hypocreaceae*, *T. virens*, *Agaricomycetes*, *Russulaceae*, and *Lactarius* were rich in healthy control monkeys (**Figure 1C**), which indicated that fungal and bacterial dysbiosis are serious occurrences in AS, and the changes of fungi showed more significant and discriminability than bacteria. There is growing research on the effects of fungi on human health, including obesity (Kim et al., 2017; Borges et al., 2018), cancer (Wang et al., 2018; Wu et al., 2019; Zhai et al., 2020), immunity, and inflammation (Leonardi et al., 2018; Li et al., 2019; Richard and Sokol, 2019). Despite the availability of new anti-fungal drugs, infections caused by pathogenic fungi continue to constitute a serious medical problem.

Studies have shown that fungal dysbiosis is correlated with the severity of IBD and, in combination with bacterial dysbiosis, could aggravate DSS-induced colitis. Fungi induce strong ecological changes to the gut microbiome, promote strong local and systemic immunological changes, and affect the immunophenotypes of the intestinal and pulmonary inflammatory responses (van Tilburg et al., 2020). These aspects indicate that fungi can modulate the severity and immunophenotype of the immune response when co-colonized with bacteria. In plant sciences, considerable communication and interaction has been recognized between fungi and bacteria (Li et al., 2019; Jiang et al., 2020; Fitzpatrick et al., 2020), while in humans, there are inadequate data.

A Chinese cohort of 84 AS patients showed that the gut microbiota was perturbed in untreated AS patients with diagnostic potential, and some AS-enriched species might be triggers of autoimmunity by molecular mimicry. More importantly, different inflammatory arthritis share some common microbial signatures (Zhou et al., 2020) such as *A. fermentans*, *B. coprophilus*, *P. distasonis*, *E. siraeum*, and *P. copri* as markers specifically enriched in the AS gut, and increased pathways including oxidative phosphorylation, LPS biosynthesis, and glycosaminoglycan degradation.

Genome-wide association studies of this highly genetic disease have implicated specific immune pathways, including the interleukin (IL)-17/IL-23 pathway, control of NF-κB activation, amino acid trimming for major histocompatibility complex (MHC) antigen presentation, and other genes controlling the CD8 and CD4 T cell subsets (Smith, 2015). Bone changes, including systemic bone loss, joint erosion, and calcaneal formation, reflect the combined effects of IL-23 and IL-17 in psoriatic arthritis (PsA) and AS (Babaie et al., 2018; Gravallese and Schett, 2018; Sieper et al., 2019). IL-17A directly or indirectly promotes osteoclast production by producing or inducing the expression of the nuclear factor receptor activator-B ligand (RANKL) (Lin et al., 2014; Ganesan and Rasool, 2017), and IL-23 multiplies the effects on osteoclasts (Ganesan and Rasool, 2017; Shukla et al., 2018). IL-17A has different effects on the maturation of precursor cells into osteoblasts, which depend on the differentiative stage of the precursor cells (Jo et al., 2018). IL-17A blockers have been shown to inhibit bone erosion, delay systemic bone loss in PsA and AS, and form mossy hyperplasia in PsA (Kampylafka et al., 2018; Samarpita et al., 2018). Therefore, in this study, we focus on this pathway to try to identify the pathogenic factors derived from microorganisms.

One suggestion for studying host–parasite interactions of *C. albicans* in rodents is that *C. albicans* is not a natural colonizer of mucosal surfaces in these animals and that the equivalent of normal colonizers in rodents is *K. pintolopesii* (Naglik et al., 2008). *C. albicans* is an important commensal fungus in the human body, and it is also the main fungal inducer of the Th17 response in humans (Bacher et al., 2019). *K. pintolopesii* can naturally colonize rodents and monkeys, so it is of great significance to further elucidate the function and pathogenicity of *K. pintolopesii* in rodents and monkeys. In this study, we found that *K. pintolopesii* would be an opportunistic and/or pathogenic inducer in AS monkeys. We also found that *K. pintolopesii* upregulated the levels of IL-23 and IL-17 in mice and could obviously damage the colonic mucosa and its integrity. The KEGG analysis of DEGs of colonic tissues showed that the pathways of *cytokine–cytokine receptor interaction*, *cell adhesion molecules* (CAMs), *natural killer cell mediated cytotoxicity*, *T-cell receptor signaling pathway*, *chemokine signaling pathway*, *osteoclast differentiation*, *phagosome*, *NF-κB signaling pathway*, *rheumatoid arthritis*, *TNF signaling pathway*, *autoimmune thyroid disease*, *inflammatory bowel disease* (IBD), *Staphylococcus aureus infection*, *intestinal immune network for IgA production*, *antigen processing and presentation*, and *B-cell receptor signaling pathway* (**Figure 4F**) were the top enriched pathways that were reportedly associated with AS, as determined in the colon of *K. pintolopesii-*infected mice. Deep analysis showed that *Gzma* and *Gzmb* would be a trigger induced by *K. pintolopesii* for the pyroptosis of neutrophils, lymphocytes, natural killer cells, and/or CD8 cytotoxic T-killer cells. DEU analysis showed that the exon E020 of *Nlrp1b*, E003 (*p* = 3.55E−17) and E005 (*p* = 1.29E−15) of *Bri3*, E001 (*p* = 5.79E−07), and E004 (*p* = 7.38E−10) of *Efhd2* would also be the influencing genes on cell pyroptosis and induce inflammation by *K. pintolopesii*.

>As the first line of defense of immune response, macrophages can maintain the homeostasis of the body by identifying and removing pathogens, killing target cells, antigen presentation, immune regulation, and other functions (Vega-Perez et al., 2021). However, excessive aggregation and activation of macrophages can also lead to tissue damage, so their homeostasis plays an important role in tissue homeostasis (Uderhardt et al., 2019; Locati et al., 2020). In our tests, we found that there were 2,622 DEGs in the RAW264.7 co-cultured with *K. pintolopesii* (**Figure 6** and **Figure S6**), which indicated that the *K. pintolopesii* infection induced violent immune responses. Deep analysis showed that genes involved inapoptosis, necroptosis and/or pyroptosis were changed (**Figure 6A** and **Figure S6**), and some of them were participate in two or all three pathways (**Figure 6A**), so we conclude that the *K. pintolopesii* induces PANoptosis of macrophage RAW264.7. Previous findings indicated that inflammatory disease-induced Th17 cells require the assistance of IL-1β and IL-23. *In vitro*, to culture the maintain tissue homeostasis Th17 cells requiring the stimulation of TGF-β and IL-6 or IL-21 (Honda and Littman, 2016; Belkaid and Harrison, 2017), and recent reports showed that SAAs could induced pathogenic Th17 cells (Lee et al., 2020).We also found the expressions of IL-17RA, IL-18RAP, IL-23A, and Il-6RA were upregulated (**Figure S6A**), and the *NF-kappaB signaling pathway*, *TNF-signaling pathway* were activated (**Figures 6B, C**); and the expressions of SAA1, IL-17A, and IL-23 were also upregulated in *K. pintolopesii* infected mice (**Figure 4**). All which indicated that the *K. pintolopesii* infected may through induced the PANoptosis of macrophage, then induces the pathogenic Th17 cells, but the action of basis and mechanism still more further studies.

In summary, our results showed that the gut mycobiota of *K. pintolopesii*, *I. perplexans*, *Saccharomycetaceae*, and *Coniochaeta* were especially enriched in AS. In addition, the microbiota of AS monkeys was characterized by increased abundance of the *Prevotalla 2* genus, *Ruminococcaceae* family, and *Clostridiales* order. Compared to healthy controls, decreased ITS2/16S biodiversity ratios and altered bacterial–fungal interkingdom networks were observed in AS monkeys. The characteristic gut mycobiota of *K. pintolopesii* induces multiple AS phenotypes in mice, such as fungal and bacterial dysbiosis, rheumatoid arthritis, inflammatory bowel disease, and varied immune responses by activation of NK cells, platelets, T cells, leukocytes, and/or B cells. All these findings imply that fungal and bacterial dysbiosis have a close link to AS and that their communication and interaction plays an important role in the autoimmune response. The exotoxin of *K. pintolopesii* may activate the IL-17RA pathway, which needs further experimental evidence. It is an interesting discovery that macrophage PANoptotic products and host TNF-a can promote the proliferation and morphological transformation of opportunistic pathogens, but more experiments are needed to clarify the specific mechanism.

### 3.1 Study limitations

Our study has some limitations. First, although we have evaluated the effects of the characteristic gut mycobiota of *K. pintolopesii* on inducing multiple AS phenotypes in normal mice, the study lacks functional tests on AS model animals. Hence, the mechanism of action of *K. pintolopesii* on AS is still unknown. Therefore, future studies should aim to use targeted strains in germ-free mice with multi-omics studies to reveal the communication and interaction mechanism of action between these AS characteristic pathogenic organisms for further validation.

## 4 Materials and methods

### 4.1 Key resources table

**Table:** 

REAGENT or RESOURCE	SOURCE	IDENTIFIER
**Antibodies**		
Anti-IL-17A	Affinity	DF6127
Anti-IL-23	Abcam	Ab45420
β-Actin (13E5)	CST	4970S
GSDMD	Affinity	#AF4012
SAA1/2 Antibody	Affinity	DF6533
GAPDH	Abcam	GR3207992-4
Goat Anti-Rabbit lgG (H + L) HRP	Affinity	S0001

**Table:** 

Chemicals		
Diaminobenzidine (DAB)	Servicebio	G1212
Goat anti-rabbit lgG (H + L) HRP	Affinity	S0001
Citrate buffer pH 6.0	Servicebio	G1202
Anhydrous ethanol	Guangzhou Guanghua Sci-Tech Co., Ltd.	1.17113.023
Penicillin–streptomycin solution	CORNING	30002304
Phosphate-buffered saline	CORNING	19117004
Fetal bovine serum	Gibco	1932595
Trimethylamine N-oxide anhydrous	Tokyo chemical industry Co., Ltd.	3EVJG-TN
0.25% Trypsin-EDTA (1×)	Gibco	2042337
DMEM basic (1×)	Gibco	8119090
Recombinant Mouse TNF alpha	Novoprotein	CF09

**Table:** 

Critical Commercial Assays		
Mouse IL-17 (Interleukin 17) ELISA Kit	Elabscience	E-EL-M0047c
Mouse TNF-alpha ELISA Kit	Proteintech	KE10002
CCK-8	Solarbio	CA1210
RNAiso Plus	TaKaRa	9108
PrimeScript™ RT reagent Kit	TaKaRa	RR047A
PrimeScript™ RT reagent Kit (RT-PCR)	TaKaRa	RR037A
TB Green™ Premix Ex Taq™ II	TaKaRa	RR820A

**Table:** 

Deposited data		
16S sequencing data	Bacterial microbiome in feces of cynomolgus monkey with spontaneous ankylosing spondylitis	PRJNA691106
ITS sequencing data	Fungal microbiome in feces of cynomolgus monkey with spontaneous ankylosing spondylitis,	PRJNA691097
RNA sequencing data	Colonic transcriptome of mice fed with *K. pintolopesii*	PRJNA690864
RNA sequencing data	Effect of *K. pintolopesii* on RAW264.7	PRJNA839846
**Software and algorithms**		
GraphPad Priam	GraphPad Software	N/A
Image J	NIH	N/A
SIMCA	Umetrics AB	N/A

### 4.2 Cell cultures

RAW264.7 cells were obtained from the National Collection of Authenticated Cell Cultures. Cells were cultured in DMEM medium with 10% fetal bovine serum as routine. All cells were grown in a humidified incubator at 37°C with 5% CO_2_.

### 4.3 CCK-8 detection

A total of 500 μl of RAW264.7 cell suspension was prepared in a 24-well plate. Plates were pre-incubated in a cell incubator for 12 h with 80 μl of 10^8^ CFU/ml *K. pintolopesii* (washed with PBS) or 80 μl of culture supernatant of *K. pintolopesii*. The control group was added with 80 μl of LB medium. Incubate in the incubator for 6 h; replace the fresh cell culture medium and add 50 μl of CCK-8 solution to each well (be careful not to generate bubbles in the well, which will affect the reading of the OD value).

### 4.4 Animals

Ten-week-old, adult, male C57BL/6 mice obtained from the Center of Laboratory Animal of Guangdong Province (SCXK [Yue] 2018-0002, SYXK [Yue] 2015-0102) were pair-housed in plastic cages in a temperature-controlled (25 ± 2°C) colony room under a 12/12-h light/dark cycle. The animals had *ad libitum* access to food and water. All experimental protocols were approved by the Center of Laboratory Animals of the Guangdong Institute of Microbiology. All efforts were made to minimize the number of animals used.

### 4.5 Histopathology and immunostaining

The arthrosis and colon tissues were removed and fixed in 4% paraformaldehyde at pH 7.4 for further pathological experiments. These tissue samples were embedded in paraffin sections after fixation, washing, dehydration, transparency, dipping in wax, and embedding. Four samples from each group were fixed in a 4% paraformaldehyde solution and prepared as paraffin sections. Sections were stained with hematoxylin and eosin (H&E). Immunostaining was performed using paraffin-embedded sections (3-μm-thick) and a two-step peroxidase-conjugated polymer technique (DAKO Envision kit, DAKO, Carpinteria, CA). Slides were observed under a light microscope.

### 4.6 Microbiome analysis

Total DNA was extracted from 250 to 500 mg of each fecal sample using the QIAamp DNA stool minikit (Qiagen, Germany). The purity and concentration of metagenomic DNA were measured with a NanoDrop 2000 spectrophotometer (Thermo Fisher Scientific, USA).

#### 4.6.1 For 16S

Construction and sequencing: after extracting the total DNA of the sample, primers were obtained according to the design of the conservative region, and the end of the primer was added with a sequencing connector. The microbial 16S rRNA genes were amplified using the forward primer 5′-ACTCCTACGGGAGGCAGCA-3′ and the reverse primer 5′-GGACTACHVGGGTWTCTAAT-3′. PCR amplification was performed, and the products were purified, quantified, and homogenized to form a sequencing library. The established library was firstly inspected by the library, and the qualified library was sequenced by Illumina HiSeq 2500. The original image data files obtained by high-throughput sequencing (Illumina HiSeq and other sequencing platforms) were analyzed and converted into original sequencing Reads by Base Calling, and the results were stored in FASTQ (fq for short) file format, which contained the sequence information of sequencing sequences (Reads) and the corresponding sequencing quality information.

#### 4.6.2 For ITS1

Construction and sequencing: after extracting the total DNA of the sample, primers were obtained according to the design of the conservative region, and the end of the primers was added with a sequencing connector. The microbial ITS genes were amplified using the forward primer ITS1F 5’-CTTGGTCATTTAGAGGAAGTAA-3’ and the reverse primer ITS2 5’-GCTGCGTTCTTCATCGATGC-3’. The rest of the steps are the same as for 16S rDNA sequencing.

#### 4.6.3 Bioinformatics analysis

Sequences of the V3–V4 region of 16S rRNA genes and ITS1 were detected using an Illumina HiSeq 2500 platform (Biomarker Technology Co., Ltd., Beijing, China). OTUs present in 50% or more of the fecal samples were identified as core OTUs. The observed species and Shannon and Simpson indices were calculated with QIIME (version 1.9.1). The abundance and diversity of the OTUs (beta diversity) were examined using principal-coordinate analysis (PCoA) with weighted or unweighted UniFrac analysis in R software. The linear discriminant analysis (LDA) and effect size analysis (LEfSe) were used with the *Kruskal–Wallis* rank sum test to detect features with significantly different abundances between assigned taxa, and the linear discriminant analysis was performed to estimate the effect size of each feature. The bacterial groups with an LDA score of 4.00 were presented as the most significantly abundant group in the indicated group.

### 4.7 RNA isolation and sequencing

RNA concentration and purity were measured using NanoDrop 2000 (Thermo Fisher Scientific, Wilmington, DE). RNA integrity was assessed using the RNA Nano 6000 Assay Kit of the Agilent Bioanalyzer 2100 system (Agilent Technologies, CA, USA). A total of 1 μg RNA per sample was used as input material for the RNA sample preparations. Sequencing libraries were generated using the NEBNext UltraTM RNA Library Prep Kit for Illumina (NEB, USA) following the manufacturer’s recommendations, and index codes were added to attribute sequences to each sample. Briefly, mRNA was purified from total RNA using poly-T oligo-attached magnetic beads. Fragmentation was carried out using divalent cations under elevated temperature in the NEBNext First Strand Synthesis Reaction Buffer (5X). First-strand cDNA was synthesized using random hexamer primers and M-MuLV reverse transcriptase. Second-strand cDNA synthesis was subsequently performed using DNA polymerase I and RNase H. Remaining overhangs were converted into blunt ends *via* exonuclease and polymerase activities. After adenylation of the 3’ ends of DNA fragments, NEBNext adaptor with a hairpin loop structure were ligated to prepare for hybridization. To select cDNA fragments of preferentially 240 bp in length, the library fragments were purified with the AMPure XP system (Beckman Coulter, Beverly, USA). Then 3 μl of USER Enzyme (NEB, USA) was used with size-selected, adaptor-ligated cDNA at 37°C for 15 min, followed by 5 min at 95°C before PCR. Then PCR was performed with Phusion High-Fidelity DNA Polymerase, Universal PCR primers, and Index (X) Primer. Finally, PCR products were purified (using the AMPure XP system), and library quality was assessed on the Agilent Bioanalyzer 2100 system. The clustering of the index-coded samples was performed on a cBot Cluster Generation System using the TruSeq PE Cluster Kit v4-cBot-HS (Illumia) according to the manufacturer’s instructions. After cluster generation, the library preparations were sequenced on an Illumina platform, and paired-end reads were generated.

### 4.8 Western blotting analysis

Briefly, global brain tissue was dissected from treated mice (purchased from Beijing HFK Bioscience Co., LTD [Certificate No: SCXK (Jing) 2014-0004]) and proteins were extracted with radioimmunoprecipitation assay (RIPA) lysis buffer (Thermo Scientific™ T-PER™ Tissue Protein Extraction Reagent, 78510). The proteins were separated by sodium dodecyl sulfate-polyacrylamide gel electrophoresis and transferred onto polyvinylidene fluoride membranes. After blocking with 5% nonfat dry milk in Tris-buffered saline (20 mM Tris–HCl, 500 mM NaCl, pH 7.4) with 0.2% Tween-20 (Aladdin, T104863), the membranes were probed with antibodies overnight at 4°C, followed by incubation with a horseradish peroxidase-conjugated goat anti-mouse (Servicebio, G2211-1-A) or goat anti-rabbit (Servicebio, G2210-2-A) IgG secondary antibody (1:2,000). Band intensity was quantified using ImageJ software (NIH).

### 4.9 Gram staining

Put the strain in nutrient broth medium, shake overnight at 37 °C, prepare OD600 = 1 bacterial solution, and dilute with nutrient broth 1:100. After dilution, add it to a 96-well polystyrene-sterilized microplate (180 μl/well) inoculate three wells. The control group was added with 20 μl of sterile nutrient broth, and the supernatant group was added with 20 μl of co-culture supernatant. In the TNF-α group, 20 μl 10,000 pg/ml TNF-α was added to bring the final concentration of TNF to 1,000 pg/ml. All groups were cultured at 37 °C for 72 h. The smear was fixed on the flame and crystal violet staining solution was added. Dyeing for 1 min and washing with water. Gram’s iodine solution was added dropwise for 1 min and washed with water. Drop the decolorizing alcohol for about 30 s, or drop the decolorizing alcohol all over the smear, pour it out immediately, and then drop the decolorating alcohol all over the smear for 10 s. Wash with water, dry, and prepare for microscopic examination.

### 4.10 Plate count

Put the strain in nutrient broth medium, shake overnight at 37 °C, prepare OD600 = 1 bacterial solution, and dilute with nutrient broth 1:100. After dilution, add it to a 96-well polystyrene-sterilized microplate (180 μl/well) and inoculate three wells. The control group was added with 20 μl of sterile nutrient broth, and the model group was added with 20 μl of co-culture supernatant for 24 h. The planktonic bacteria stimulated by the supernatants of the two groups were collected, diluted by 10^6^ times, and then 10 μl of the supernatant was spread on the broth agar plate, which was placed in a constant temperature incubator at 37 °C for 24 h and counted.

### 4.11 Statistical analysis

All data are described as the means ± standard deviations (SD) of at least three independent experiments. The significant differences between treatments were analyzed using one-way analysis of variance (ANOVA) or the T test at *p<*0.05. The Statistical Package for the Social Sciences (SPSS, Abacus Concepts, Berkeley, CA, USA) and Prism 8 (GraphPad, San Diego, CA, USA) software were used for all statistical analyses.

## Data availability statement

The datasets presented in this study can be found in online repositories. The names of the repository/repositories and accession number(s) can be found in the article/**Supplementary material**.

## Ethics statement

The animal study was reviewed and approved by Guangdong Institute of Microbiology.

## Author contributions

DC, JW, and MC designed this study. DC and YG wrote the manuscript. HJ, HZ, and YG collected animal physiological data and fecal samples, extracted ruminal DNA, did the physiological and biochemical indexes measurement. DC, MC, and YG carried out the computational analyses. XT did the western bolting analysis. YW did the animal and cell experiments. HZ and HJ did the histopathology and immunostaining of animal. DC, JW, and MC reviewed this manuscript and offer all the necessary research start-up fund and experimental platform. All authors listed have made a substantial, direct, and intellectual contribution to the work and approved it for publication.

## Supplementary material

Supplementary Table 1 KEGG pathway enrichment analysis of differentially expressed genes in *K. pintolopesii* induces multiple AS phenotypes mice

Supplementary Figure S1 Altered mycobiota biodiversity and composition in AS and health control monkeys. (A) Global composition of fungal microbiota at the genus levels showing in heatmap.

Supplementary Figure S2 Altered mycobiota biodiversity and composition in AS and health control monkeys. Global composition of fungal microbiota at the phylum **(A)**, class **(B)**, order **(C)**, family **(D)**, genus **(F)**, species **(G)** levels showing in column diagram.

Supplementary Figure S3 Altered bacterial biodiversity and composition in AS and health control monkeys. **(A)** Global composition of bacterial microbiota at the genus levels showing in heatmap.

Supplementary Figure S4 Altered bacterial biodiversity and composition in AS and health control monkeys. Global composition of bacterial microbiota at the phylum **(A)**, class **(B)**, order **(C)**, family **(D)**, genus **(E)**, species **(F)** levels showing in column diagram.

Supplementary Figure S5 The GO classification of differentially expressed genes of *K. pintolopesii*-induced colon of mice.

Supplementary Figure S6 Gene Set Enrichment Analysis (GSEA) using DEXSeq (version 1.12.2, default parameters) of DEGs in *K. pintolopesii* induces mice. **(A)** innate immune response. **(B)** inflammatory response. **(C)** integral component of plasma membrane. **(D)** transcription factor complex. **(E)** antigen binding. **(F)** carbohydrate binding.

Supplementary Figure S7 The RNA sequencing of RAW264.7 co-cultured with K. pintolopesii showed that about 2622 mRNAs changes in expression, with 1016 mRNAs up-regulated and 1606 down-regulated (fold change [FC]≥1.5 and FDR<0.05, A); KEGG analysis of differentially up-expressed 1016 genes **(B)**; KEGG analysis of differentially down-expressed 1606 genes.

Supplementary Figure S8 The GO classification of differentially expressed genes of RAW264.7 co-cultured with *K. pintolopesii.*

## References

[B1] Amezcua Vesely M. C. Pallis P. Bielecki P. Low J. S. Zhao J. Harman C. C. D.. (2019). Effector TH17 cells give rise to long-lived TRM cells that are essential for an immediate response against bacterial infection. Cell 178 (5), 1176–1188. doi: 10.1016/j.cell.2019.07.032 31442406 PMC7057720

[B2] Avramidou A. Kroczek C. Lang C. Schuh W. Jack H. M. Mielenz D. (2007). The novel adaptor protein swiprosin-1 enhances BCR signals and contributes to BCR-induced apoptosis. Cell Death Differ 14 (11), 1936–1947. doi: 10.1038/sj.cdd.4402206 17673920

[B3] Babaie F. Hasankhani M. Mohammadi H. Safarzadeh E. Rezaiemanesh A. Salimi R.. (2018). The role of gut microbiota and IL-23/IL-17 pathway in ankylosing spondylitis immunopathogenesis: New insights and updates. Immunol. Lett. 196, 52–62. doi: 10.1016/j.imlet.2018.01.014 29409751

[B4] Bacher P. Hohnstein T. Beerbaum E. Röcker M. Blango M. G. Kaufmann S.. (2019). Human anti-fungal Th17 immunity and pathology rely on cross-reactivity against candida albicans. Cell 176 (6), 1340–1355.e15. doi: 10.1016/j.cell.2019.01.041 30799037

[B5] Belkaid Y. Harrison O. J. (2017). Homeostatic immunity and the microbiota. Immunity 46 (4), 562–576. doi: 10.1016/j.immuni.2017.04.008 28423337 PMC5604871

[B6] Borges F. M. de Paula T. O. Sarmiento M. de Oliveira M. G. Pereira M. Toledo I. V.. (2018). Fungal diversity of human gut microbiota among eutrophic, overweight, and obese individuals based on aerobic culture-dependent approach. Curr. Microbiol. 75 (6), 726–735. doi: 10.1007/s00284-018-1438-8 29368026

[B7] Braun J. Sieper J. (2007). Ankylosing spondylitis. Lancet 369 (9570), 1379–1390. doi: 10.1016/S0140-6736(07)60635-7 17448825

[B8] Ciccia F. Ferrante A. Triolo G. (2016). Intestinal dysbiosis and innate immune responses in axial spondyloarthritis. Curr. Opin. Rheumatol. 28 (4), 352–358. doi: 10.1097/BOR.0000000000000296 27214393

[B9] Ciccia F. Guggino G. Rizzo A. Alessandro R. Luchetti M. M. Milling S.. (2017). Dysbiosis and zonulin upregulation alter gut epithelial and vascular barriers in patients with ankylosing spondylitis. Ann. Rheumatic Dis. 76 (6), 1123–1132. doi: 10.1136/annrheumdis-2016-210000 PMC6599509 28069576

[B10] Costello M. Elewaut D. Kenna T. J. Brown M. A. (2013). Microbes, the gut and ankylosing spondylitis. Arthritis Res. Ther. 15 (3), 214. doi: 10.1186/ar4228 23750937 PMC4060176

[B11] Fitzpatrick C. R. Salas-Gonzalez I. Conway J. M. Finkel O. M. Gilbert S. Russ D.. (2020). The plant microbiome: From ecology to reductionism and beyond. Annu. Rev. Microbiol. 74, 81–100. doi: 10.1146/annurev-micro-022620-014327 32530732

[B12] Fragoulis G. E. Liava C. Daoussis D. Akriviadis E. Garyfallos A. Dimitroulas T. (2019). Inflammatory bowel diseases and spondyloarthropathies: From pathogenesis to treatment. World J. Gastroenterol. 25 (18), 2162–2176. doi: 10.3748/wjg.v25.i18.2162 31143068 PMC6526158

[B13] Ganesan R. Rasool M. (2017). Interleukin 17 regulates SHP-2 and IL-17RA/STAT-3 dependent Cyr61, IL-23 and GM-CSF expression and RANKL mediated osteoclastogenesis by fibroblast-like synoviocytes in rheumatoid arthritis. Mol. Immunol. 91, 134–144. doi: 10.1016/j.molimm.2017.09.003 28898718

[B14] Gill T. Asquith M. Brooks S. R. Rosenbaum J. T. Colbert R. A. (2018). Effects of HLA-B27 on gut microbiota in experimental spondyloarthritis implicate an ecological model of dysbiosis. Arthritis Rheumatol 70 (4), 555–565. doi: 10.1002/art.40405 29287307 PMC6101666

[B15] Gravallese E. M. Schett G. (2018). Effects of the IL-23-IL-17 pathway on bone in spondyloarthritis. Nat. Rev. Rheumatol 14 (11), 631–640. doi: 10.1038/s41584-018-0091-8 30266977

[B16] Honda K. Littman D. R. (2016). The microbiota in adaptive immune homeostasis and disease. Nature 535 (7610), 75–84. doi: 10.1038/nature18848 27383982

[B17] Huang R. Li F. Zhou Y. Zeng Z. He X. Fang L.. (2020). Metagenome-wide association study of the alterations in the intestinal microbiome composition of ankylosing spondylitis patients and the effect of traditional and herbal treatment. J. Med. Microbiol. 69 (6), 797–805. doi: 10.1099/jmm.0.001107 31778109 PMC7451032

[B18] Jia H. Chen M. Cai Y. Luo X. Hou G. Li Y.. (2022). A new and spontaneous animal model for ankylosing spondylitis is found in cynomolgus monkeys. Arthritis Res. Ther. 24 (1), 1. doi: 10.1186/s13075-021-02679-5 34980262 PMC8722021

[B19] Jiang C. Hei R. Yang Y. Zhang S. Wang Q. Wang W.. (2020). An orphan protein of fusarium graminearum modulates host immunity by mediating proteasomal degradation of TaSnRK1alpha. Nat. Commun. 11 (1), 4382. doi: 10.1038/s41467-020-18240-y 32873802 PMC7462860

[B20] Jiang L. Wang Y. J. Zhao J. Uehara M. Hou Q. Kasinath V.. (2020). Direct tumor killing and immunotherapy through anti-SerpinB9 therapy. Cell 183 (5), 1219–1233.e18. doi: 10.1016/j.cell.2020.10.045 33242418 PMC7927154

[B21] Jostins L. Ripke S. Weersma R. K. Duerr R. H. McGovern D. P. Hui K. Y.. (2012). Host–microbe interactions have shaped the genetic architecture of inflammatory bowel disease. Nature 491 (7422), 119–124. doi: 10.1038/nature11582 23128233 PMC3491803

[B22] Jo S. Wang S. E. Lee Y. L. Kang S. Lee B. Han J.. (2018). IL-17A induces osteoblast differentiation by activating JAK2/STAT3 in ankylosing spondylitis. Arthritis Res. Ther. 20 (1), 115. doi: 10.1186/s13075-018-1582-3 29880011 PMC5992730

[B23] Kampylafka E. D’Oliveira I. Linz C. Lerchen V. Stemmler F. Simon D.. (2018). Resolution of synovitis and arrest of catabolic and anabolic bone changes in patients with psoriatic arthritis by IL-17A blockade with secukinumab: results from the prospective PSARTROS study. Arthritis Res. Ther. 20 (1), 153. doi: 10.1186/s13075-018-1653-5 30053825 PMC6063019

[B24] Kim D. H. Kim H. Jeong D. Kang I. B. Chon J. W. Kim H. S.. (2017). Kefir alleviates obesity and hepatic steatosis in high-fat diet-fed mice by modulation of gut microbiota and mycobiota: targeted and untargeted community analysis with correlation of biomarkers. J. Nutr. Biochem. 44, 35–43. doi: 10.1016/j.jnutbio.2017.02.014 28384519

[B25] Klingberg E. Magnusson M. K. Strid H. Deminger A. Ståhl A. Sundin J.. (2019). A distinct gut microbiota composition in patients with ankylosing spondylitis is associated with increased levels of fecal calprotectin. Arthritis Res. Ther. 21 (1), 248. doi: 10.1186/s13075-019-2018-4 31771630 PMC6880506

[B26] Koning F. Thomas R. Rossjohn J. Toes R. E. (2015). Coeliac disease and rheumatoid arthritis: similar mechanisms, different antigens. Nat. Rev. Rheumatol. 11 (8), 450–461. doi: 10.1038/nrrheum.2015.59 25986717

[B27] Kroczek C. Lang C. Brachs S. Grohmann M. Dutting S. Schweizer A.. (2010). Swiprosin-1/EFhd2 controls b cell receptor signaling through the assembly of the b cell receptor, syk, and phospholipase c gamma2 in membrane rafts. J. Immunol. 184 (7), 3665–3676. doi: 10.4049/jimmunol.0903642 20194721

[B28] Lee J. Y. Hall J. A. Kroehling L. Wu L. Najar T. Nguyen H. H.. (2020). Serum amyloid a proteins induce pathogenic Th17 cells and promote inflammatory disease. Cell 183 (7), 2036–2039. doi: 10.1016/j.cell.2020.12.008 33357400 PMC7891798

[B29] Leonardi I. Li X. Semon A. Li D. Doron I. Putzel G.. (2018). CX3CR1(+) mononuclear phagocytes control immunity to intestinal fungi. Science 359 (6372), 232–236. doi: 10.1126/science.aao1503 29326275 PMC5805464

[B30] Li M. Dai B. Tang Y. Lei L. Li N. Liu C.. (2019). Altered bacterial-fungal interkingdom networks in the guts of ankylosing spondylitis patients. mSystems 4 (2). doi: 10.1128/mSystems.00176-18 PMC6435815 30944880

[B31] Li X. V. Leonardi I. Iliev I. D. (2019). Gut mycobiota in immunity and inflammatory disease. Immunity 50 (6), 1365–1379. doi: 10.1016/j.immuni.2019.05.023 31216461 PMC6585451

[B32] Lin D. Li L. Sun Y. Wang W. Wang X. Ye Y.. (2014). IL-17 regulates the expressions of RANKL and OPG in human periodontal ligament cells *via* TRAF6/TBK1-JNK/NF-kappaB pathways. Immunology. 144 (3), 472–485. doi: 10.1111/imm.12395 25263088 PMC4557684

[B33] Li Z. Ye X. Liu M. Xia C. Zhang L. Luo X.. (2019). A novel outer membrane beta-1,6-glucanase is deployed in the predation of fungi by myxobacteria. ISME J. 13 (9), 2223–2235. doi: 10.1038/s41396-019-0424-x 31065029 PMC6776036

[B34] Locati M. Curtale G. Mantovani A. (2020). Diversity, mechanisms, and significance of macrophage plasticity. Annu. Rev. Pathol. 15, 123–147. doi: 10.1146/annurev-pathmechdis-012418-012718 31530089 PMC7176483

[B35] Luoma A. M. Suo S. Williams H. L. Sharova T. Sullivan K. Manos M.. (2020). Molecular pathways of colon inflammation induced by cancer immunotherapy. Cell 182 (3), 655–671.e22. doi: 10.1016/j.cell.2020.06.001 32603654 PMC7415717

[B36] McGonagle D. Stockwin L. Isaacs J. Emery P. (2001). An enthesitis based model for the pathogenesis of spondyloarthropathy Additive effects of microbial adjuvant and biomechanical factors at disease sites. J. Rheumatol. 28 (10), 2155. doi: 10.1016/S1297-319X(01)00304-9 11669149

[B37] Naglik J. R. Fidel P. J. Odds F. C. (2008). Animal models of mucosal candida infection. FEMS Microbiol. Lett. 283 (2), 129–139. doi: 10.1111/j.1574-6968.2008.01160.x 18422625 PMC3244615

[B38] Nakamoto N. Sasaki N. Aoki R. Miyamoto K. Suda W. Teratani T.. (2019). Gut pathobionts underlie intestinal barrier dysfunction and liver T helper 17 cell immune response in primary sclerosing cholangitis. Nat. Microbiol. 4 (3), 492–503. doi: 10.1038/s41564-018-0333-1 30643240

[B39] Reid G. Younes J. A. van der Mei H. C. Gloor G. B. Knight R. Busscher H. J. (2011). Microbiota restoration: natural and supplemented recovery of human microbial communities. Nat. Rev. Microbiol. 9 (1), 27–38. doi: 10.1038/nrmicro2473 21113182

[B40] Richard M. L. Sokol H. (2019). The gut mycobiota: insights into analysis, environmental interactions and role in gastrointestinal diseases. Nat. Rev. Gastroenterol. Hepatol. 16 (6), 331–345. doi: 10.1038/s41575-019-0121-2 30824884

[B41] Rooks M. G. Garrett W. S. (2016). Gut microbiota, metabolites and host immunity. Nat. Rev. Immunol. 16 (6), 341–352. doi: 10.1038/nri.2016.42 27231050 PMC5541232

[B42] Samarpita S. Doss H. M. Ganesan R. Rasool M. (2018). Interleukin 17 under hypoxia mimetic condition augments osteoclast mediated bone erosion and expression of HIF-1alpha and MMP-9. Cell Immunol. 332, 39–50. doi: 10.1016/j.cellimm.2018.07.005 30029761

[B43] Scher J. U. Littman D. R. Abramson S. B. (2016). Review: Microbiome in inflammatory arthritis and human rheumatic diseases. Arthritis Rheumatol. 68 (1), 35–45. doi: 10.1002/art.39259 26331579 PMC4789258

[B44] Shukla P. Mansoori M. N. Singh D. (2018). Efficacy of anti-IL-23 monotherapy versus combination therapy with anti-IL-17 in estrogen deficiency induced bone loss conditions. Bone 110, 84–95. doi: 10.1016/j.bone.2018.01.027 29414600

[B45] Sieper J. Poddubnyy D. (2017). Axial spondyloarthritis. Lancet 390 (10089), 73–84. doi: 10.1016/S0140-6736(16)31591-4 28110981

[B46] Sieper J. Poddubnyy D. Miossec P. (2019). The IL-23-IL-17 pathway as a therapeutic target in axial spondyloarthritis. Nat. Rev. Rheumatol 15 (12), 747–757. doi: 10.1038/s41584-019-0294-7 31551538

[B47] Simone D. Al Mossawi M. H. Bowness P. (2018). Progress in our understanding of the pathogenesis of ankylosing spondylitis. Rheumatology 57 (suppl_6), vi4–vi9. doi: 10.1093/rheumatology/key001 30445483 PMC6238220

[B48] Smith J. A. (2015). Update on ankylosing spondylitis: current concepts in pathogenesis. Curr. Allergy Asthma Rep. 15 (1), 489. doi: 10.1007/s11882-014-0489-6 25447326

[B49] Uderhardt S. Martins A. J. Tsang J. S. Lammermann T. Germain R. N. (2019). Resident macrophages cloak tissue microlesions to prevent neutrophil-driven inflammatory damage. Cell 177 (3), 541–555.e17. doi: 10.1016/j.cell.2019.02.028 30955887 PMC6474841

[B50] Van Praet L. Jans L. Carron P. Jacques P. Glorieus E. Colman R.. (2014). Degree of bone marrow oedema in sacroiliac joints of patients with axial spondyloarthritis is linked to gut inflammation and male sex: results from the GIANT cohort. Ann. Rheumatic Dis. 73 (6), 1186–1189. doi: 10.1136/annrheumdis-2013-203854 24276368

[B51] van Tilburg B. E. Pettersen V. K. Gutierrez M. W. Laforest-Lapointe I. Jendzjowsky N. G. Cavin J. B.. (2020). Intestinal fungi are causally implicated in microbiome assembly and immune development in mice. Nat. Commun. 11 (1), 2577. doi: 10.1038/s41467-020-16431-1 32444671 PMC7244730

[B52] Vega-Perez A. Villarrubia L. H. Godio C. Gutierrez-Gonzalez A. Feo-Lucas L. Ferriz M.. (2021). Resident macrophage-dependent immune cell scaffolds drive anti-bacterial defense in the peritoneal cavity. Immunity 54 (11), 2578–2594.e5. doi: 10.1016/j.immuni.2021.10.007 34717795

[B53] Wang T. Fan C. Yao A. Xu X. Zheng G. You Y.. (2018). The adaptor protein CARD9 protects against colon cancer by restricting mycobiota-mediated expansion of myeloid-derived suppressor cells. Immunity 49 (3), 504–514.e4. doi: 10.1016/j.immuni.2018.08.018 30231984 PMC6880241

[B54] Watad A. Bridgewood C. Russell T. Marzo-Ortega H. Cuthbert R. McGonagle D. (2018). The early phases of ankylosing spondylitis: Emerging insights from clinical and basic science. Front. Immunol. 9, 2668. doi: 10.3389/fimmu.2018.02668 30505307 PMC6250731

[B55] Wu M. Li J. An Y. Li P. Xiong W. Li J.. (2019). Chitooligosaccharides prevents the development of colitis-associated colorectal cancer by modulating the intestinal microbiota and mycobiota. Front. Microbiol. 10, 2101. doi: 10.3389/fmicb.2019.02101 31620100 PMC6759605

[B56] Wu H. Liu G. Li C. Zhao S. (2003). bri3, a novel gene, participates in tumor necrosis factor-alpha-induced cell death. Biochem. Biophys. Res. Commun. 311 (2), 518–524. doi: 10.1016/j.bbrc.2003.10.038 14592447

[B57] Xiao G. Wang X. Wang J. Zu L. Cheng G. Hao M.. (2015). CXCL16/CXCR6 chemokine signaling mediates breast cancer progression by pERK1/2-dependent mechanisms. Oncotarget 6 (16), 14165–14178. doi: 10.18632/oncotarget.3690 25909173 PMC4546458

[B58] Xu Y. Y. Tan X. He Y. T. Zhou Y. Y. He X. H. Huang R. Y. (2016). Role of gut microbiome in ankylosing spondylitis: an analysis of studies in literature. Discovery Med. 22 (123), 361–370. 28147218

[B59] Zhai B. Ola M. Rolling T. Tosini N. L. Joshowitz S. Littmann E. R.. (2020). High-resolution mycobiota analysis reveals dynamic intestinal translocation preceding invasive candidiasis. Nat. Med. 26 (1), 59–64. doi: 10.1038/s41591-019-0709-7 31907459 PMC7005909

[B60] Zhang Z. Zhang Y. Xia S. Kong Q. Li S. Liu X.. (2020). Gasdermin e suppresses tumour growth by activating anti-tumour immunity. Nature 579 (7799), 415–420. doi: 10.1038/s41586-020-2071-9 32188940 PMC7123794

[B61] Zhou C. Zhao H. Xiao X. Chen B. Guo R. Wang Q.. (2020). Metagenomic profiling of the pro-inflammatory gut microbiota in ankylosing spondylitis. J. Autoimmun. 107, 102360. doi: 10.1016/j.jaut.2019.102360 31806420

