# Supplementary material for: Immune activation of characteristic gut mycobiota Kazachstania pintolopesii on IL-23/IL-17R signaling in ankylosing spondylitis
Source: Front Cell Infect Microbiol. 2022 Dec 20;12:1035366. doi: 10.3389/fcimb.2022.1035366 (PMC9808786; doi:10.3389/fcimb.2022.1035366)
Supplement: Supplementary Table 1 — KEGG pathway enrichment analysis of differentially expressed genes in K. pintolopesii induces multiple AS phenotypes mice [file Table_1.docx]

**Table S1** KEGG pathway enrichment analysis of differentially expressed genes in *K. pintolopesii* induces multiple AS phenotypes mice

| **Description** | ***p*-value** | **geneID** | **Gene number** | **Contained** |
| --- | --- | --- | --- | --- |
| Cytokine-cytokine receptor interaction | 1.78E-10 | I*l12rb1; Cd40; Il12rb2; Cxcl16; Ccl9; Ifnar2; Ltb; Csf1r; Il18rap; Il18r1; Il2ra; Bmp2; Il1b; Pdgfc; Csf3r; Cxcl9; Ccr9; Cd27; Il21r; Il4ra; Il2rg; Pdgfd; Il10ra; Crlf2; Ccl5; Ccl2; Inhbb; Il18; Cxcr6; Ccr2; Cxcr3; Cx3cr1; Cxcl2; Xcr1; Il2rb; Csf2rb; Csf2rb2; Ccl28; Ccr5* | 39 | up&down |
| Cell adhesion molecules (CAMs) | 1.42E-13 | *Itgb2; Itgb7; Cd40; Cd86; Cd4; Cd6; Icos; Ctla4; Cd28; Pdcd1; Ptprc; Itga4; Cd2; Vcam1; Itgam; Itgal; Cd226; H2-Aa; Icam1; H2-DMa; Itga9; Selplg; Spn; Cd8b1; Cd8a; H2-T3; H2-Q5; H2-Q7; H2-Eb1; Tigit; Gm8909; H2-Q6; H2-Ab1; H2-DMb1; H2-Q2; Mus_musculus_newGene_3650* | 36 | down |
| Natural killer cell mediated cytotoxicity | 4.08E-16 | *Itgb2; Lck; Lcp2; Ptpn6; Cd244a; Cd247; Vav2; Cd48; Gzmb; Fyn; Pik3r5; Ppp3cc; Ifnar2; Zap70; Klrk1; Klrd1; Klrc1; Tyrobp; Lat; Itgal; Nfatc1; Rac2; Vav3; Prf1; Icam1; Pik3cd; Prkcb; Sh3bp2; Fcer1g; Fcgr4; Mus_musculus_newGene_6834; Mus_musculus_newGene_6846; Mus_musculus_newGene_6848; Mus_musculus_newGene_6901; Mus_musculus_newGene_6925* | 35 | up&down |
| Herpes simplex infection | 3.45E-10 | *Irf9; Ifnar2; Eif2ak2; C3; Cd74; Irf7; Sp100; Traf1; Ifih1; Il1b; Oas1b; Oas3; Oas2; Ifit1; Ccl5; Ccl2; H2-Aa; H2-DMa; Ddx58; Tlr9; Pilra; Jun; Oas1a; Socs3; H2-T3; H2-Q5; H2-Q7; H2-Eb1; Oas1g; Gm8909; H2-Q6; H2-Ab1; Ifit1bl1; H2-DMb1; H2-Q2* | 35 | up&down |
| T cell receptor signaling pathway | 5.38E-18 | *Lck; Cd3g; Lcp2; Ptpn6; Cd247; Vav2; Fyn; Itk; Pik3r5; Map3k14; Ppp3cc; Cd4; Nfkbie; Icos; Ctla4; Cd28; Zap70; Pdcd1; Ptprc; Prkcq; Rasgrp1; Lat; Cd3e; Cd3d; Nfatc1; Vav3; Card11; Pik3cd; Grap2; Jun; Cd8b1; Cd8a; Gm49320; Mus_musculus_newGene_3650* | 34 | up&down |
| Measles | 7.48E-12 | *Cd3g; Irf9; Fyn; Pik3r5; Ifnar2; Mx2; Eif2ak2; Irf7; Cd28; Il2ra; Prkcq; Ifih1; Il1b; Adar; Oas1b; Msn; Il2rg; Cd3e; Cd3d; Oas3; Oas2; Pik3cd; Ddx58; Tlr9; Oas1a; Oas1g; Il2rb; Hspa1a; Mus_musculus_newGene_3650; Mus_musculus_newGene_6834; Mus_musculus_newGene_6846; Mus_musculus_newGene_6848; Mus_musculus_newGene_6901; Mus_musculus_newGene_6925* | 34 | up&down |
| Influenza A | 1.06E-10 | *Irf9; Rsad2; Pik3r5; Ciita; Ifnar2; Mx2; Eif2ak2; Irf7; Ifih1; Il1b; Adar; Oas1b; Oas3; Oas2; Nlrp3; Ccl5; Ccl2; H2-Aa; Icam1; H2-DMa; Il18; Pik3cd; Ddx58; Jun; Oas1a; Socs3; H2-Eb1; Oas1g; H2-Ab1; H2-DMb1; Hspa1a; Gm49320* | 32 | up&down |
| Chemokine signaling pathway | 6.00E-10 | *Hck; Vav2; Ncf1; Cxcl16; Ccl9; Dock2; Itk; Pik3r5; Fgr; Cxcl9; Ccr9; Was; Adcy7; Rac2; Vav3; Ccl5; Ccl2; Prex1; Pik3cd; Elmo1; Grk3; Gng2; Cxcr6; Ccr2; Cxcr3; Cx3cr1; Prkcb; Cxcl2; Arrb2; Xcr1; Ccl28; Ccr5* | 32 | up&down |
| Osteoclast differentiation | 3.95E-13 | *Lck; Spi1; Irf9; Lcp2; Cybb; Ncf1; Fyn; Pik3r5; Map3k14; Ppp3cc; Ifnar2; Csf1r; Il1b; Tyrobp; Nfatc1; Sirpa; Socs1; Pik3cd; Jun; Socs3; Pirb; Fcgr4; Fcgr3; Gm49339; Ncf4; Gm14548; Gm15922; Pira2; Gm49320; Lilrb4a; Mus_musculus_newGene_4127* | 31 | up&down |
| Staphylococcus aureus infection | 1.04E-13 | *Itgb2; C3; Itgam; Itgal; H2-Aa; C1qa; C1qc; C1qb;I cam1; H2-DMa; C1s1; C3ar1; Selplg; C5ar1; Ptafr; Fcgr4; Fcgr3; H2-Eb1; H2-Ab1; H2-DMb1; Mus_musculus_newGene_3650; Mus_musculus_newGene_6834; Mus_musculus_newGene_6846; Mus_musculus_newGene_6848; Mus_musculus_newGene_6901; Mus_musculus_newGene_6925* | 26 | up&down |
| Viral myocarditis | 2.06E-10 | *Itgb2; Cd40; Fyn; Cd86; Cd28; Itgal; Rac2; H2-Aa; Prf1; Icam1; H2-DMa; H2-T3; H2-Q5; H2-Q7; H2-Eb1; Gm8909; H2-Q6; H2-Ab1; H2-DMb1; H2-Q2; Mus_musculus_newGene_3650; Mus_musculus_newGene_6834; Mus_musculus_newGene_6846; Mus_musculus_newGene_6848; Mus_musculus_newGene_6901; Mus_musculus_newGene_6925* | 26 | up&down |
| Leishmaniasis | 1.06E-11 | *Itgb2; Ptpn6; Ncf1; Nos2; C3; Itga4; Il1b; Itgam; Ptgs2; H2-Aa; H2-DMa; Jun; Prkcb; Fcgr4; Fcgr3; H2-Eb1; Ncf4; H2-Ab1; H2-DMb1; Mus_musculus_newGene_6834; Mus_musculus_newGene_6846; Mus_musculus_newGene_6848; Mus_musculus_newGene_6901; Mus_musculus_newGene_6925* | 24 | up&down |
| Hematopoietic cell lineage | 4.39E-09 | *Cd3g; Cd44; Cd4; Csf1r; Cd5; Cd7; Il2ra; Itga4; Il1b; Cd2; Csf3r; Il4ra; Itgam; Cd3e; Cd3d; Cd8b1; Cd8a; H2-Eb1; Mus_musculus_newGene_6834; Mus_musculus_newGene_6846; Mus_musculus_newGene_6848; Mus_musculus_newGene_6901; Mus_musculus_newGene_6925* | 23 | up&down |
| Autoimmune thyroid disease | 5.24E-09 | *Gzmb; Cd40; Cd86; Ctla4; Cd28; H2-Aa; Prf1; H2-DMa; H2-T3; H2-Q5; H2-Q7; H2-Eb1; Gm8909; H2-Q6; H2-Ab1; H2-DMb1; H2-Q2; Mus_musculus_newGene_3650; Mus_musculus_newGene_6834; Mus_musculus_newGene_6846; Mus_musculus_newGene_6848; Mus_musculus_newGene_6901; Mus_musculus_newGene_6925* | 23 | up&down |
| Allograft rejection | 1.72E-09 | *Gzmb; Cd40; Cd86; Cd28; H2-Aa; Prf1; H2-DMa; H2-T3; H2-Q5; H2-Q7; H2-Eb1; Gm8909; H2-Q6; H2-Ab1; H2-DMb1; H2-Q2; Mus_musculus_newGene_3650; Mus_musculus_newGene_6834; Mus_musculus_newGene_6846; Mus_musculus_newGene_6848; Mus_musculus_newGene_6901; Mus_musculus_newGene_6925* | 22 | up&down |
| Antigen processing and presentation | 9.89E-09 | *Lgmn; Ciita; Cd4; Cd74; Klrd1; Klrc1; H2-Aa; H2-DMa; Cd8b1; Cd8a; H2-T3; H2-Q5; H2-Q7; H2-Eb1; Gm8909; H2-Q6; H2-Ab1; H2-DMb1; H2-Q2; Hspa1a; Mus_musculus_newGene_3650* | 21 | down |
| Inflammatory bowel disease (IBD) | 4.69E-11 | *Il12rb1; Tbx21; Il12rb2; Il18rap; Il18r1; Il1b; Il21r; Il4ra; Il2rg; Rora; Nfatc1; H2-Aa; H2-DMa; Il18; Jun; H2-Eb1; Stat4; H2-Ab1; H2-DMb1; Mus_musculus_newGene_3650* | 20 | up&down |
| Intestinal immune network for IgA production | 1.76E-09 | *Itgb7; Cd40; Map3k14; Cd86; Icos; Cd28; Itga4; Ccr9; H2-Aa; H2-DMa; H2-Eb1; H2-Ab1; Ccl28; H2-DMb1; Mus_musculus_newGene_3650; Mus_musculus_newGene_6834; Mus_musculus_newGene_6846; Mus_musculus_newGene_6848; Mus_musculus_newGene_6901; Mus_musculus_newGene_6925* | 20 | up&down |
| Graft-versus-host disease | 3.85E-10 | *Gzmb; Cd86; Cd28; Il1b; Klrd1; Klrc1; H2-Aa; Prf1; H2-DMa; H2-T3; H2-Q5; H2-Q7; H2-Eb1; Gm8909; H2-Q6; H2-Ab1; H2-DMb1; H2-Q2; Mus_musculus_newGene_3650* | 19 | down |
| Primary immunodeficiency | 1.01E-08 | *Lck; Cd40; Ciita; Cd4; Icos; Zap70; Ptprc; Il2rg; Cd3e; Cd3d; Cd8b1; Cd8a; Mus_musculus_newGene_6834; Mus_musculus_newGene_6846; Mus_musculus_newGene_6848; Mus_musculus_newGene_6901; Mus_musculus_newGene_6925* | 17 | up&down |
