# Supplementary figures and images for: Immune activation of characteristic gut mycobiota Kazachstania pintolopesii on IL-23/IL-17R signaling in ankylosing spondylitis
Source: Front Cell Infect Microbiol. 2022 Dec 20;12:1035366. doi: 10.3389/fcimb.2022.1035366 (PMC9808786; doi:10.3389/fcimb.2022.1035366)

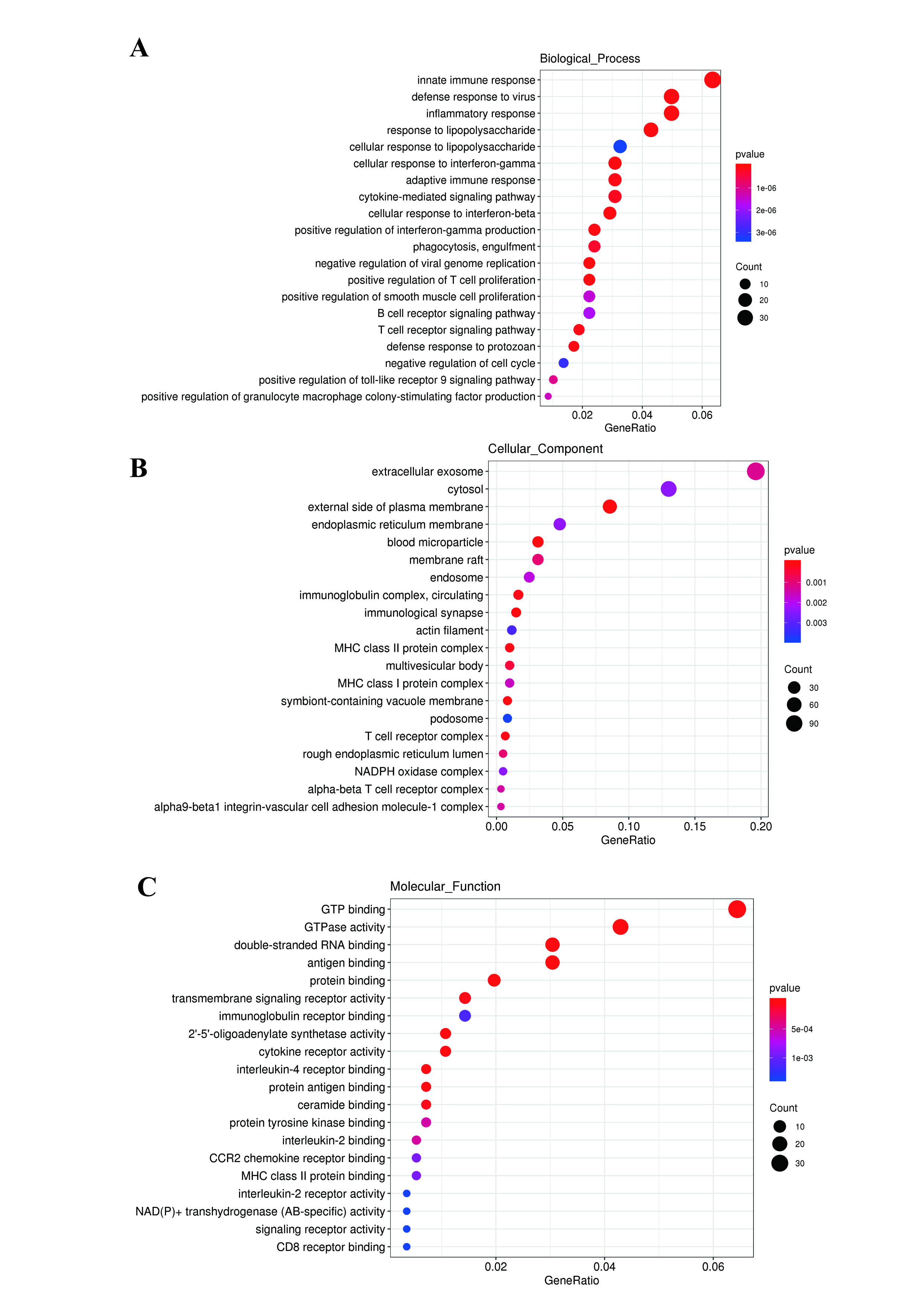

Supplement: Supplementary Figure S5 — The GO classification of differentially expressed genes of K. pintolopesii-induced colon of mice. [file Image_5.tif]

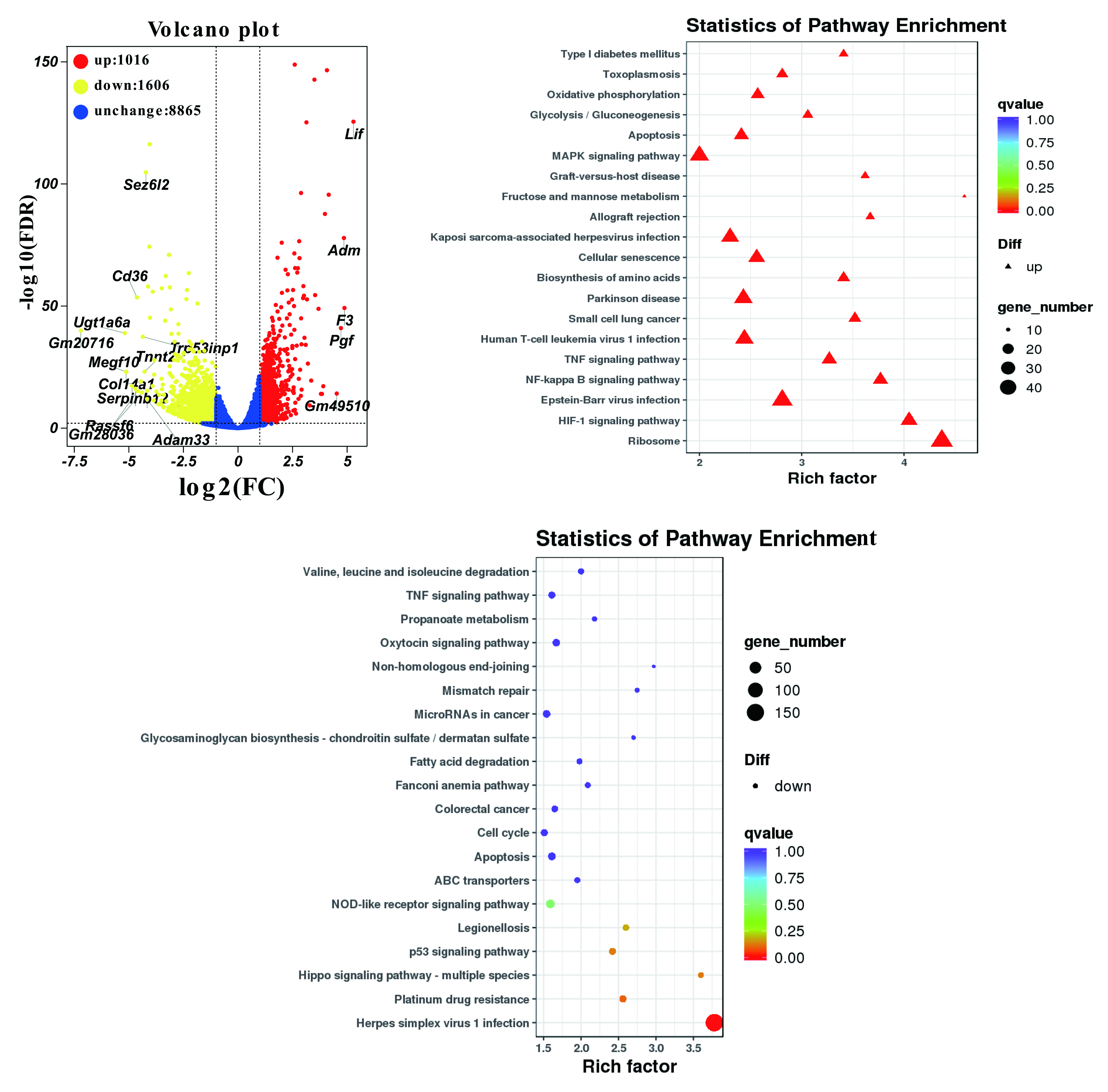

Supplement: Supplementary Figure S7 — The RNA sequencing of RAW264.7 co-cultured with K. pintolopesii showed that about 2622 mRNAs changes in expression, with 1016 mRNAs up-regulated and 1606 down-regulated (fold change [FC]≥1.5 and FDR<0.05, A); KEGG analysis of differentially up-expressed 1016 genes (B); KEGG analysis of differentially down-expressed 1606 genes. [file Image_7.tif]
